# Integrated Assessment of Wall Shear Stress‐Related Hemodynamic Parameters in Abdominal Aortic Aneurysms: A Retrospective Cross‐Sectional Study on Ruptured Cases

**DOI:** 10.1002/cnm.70153

**Published:** 2026-03-20

**Authors:** Onur Mutlu, Rahib A. Khan, Huseyin E. Salman, Ayman El‐Menyar, Mehmet M. Yavuz, Muhammad E. H. Chowdhury, Hassan Al‐Thani, Huseyin C. Yalcin

**Affiliations:** ^1^ Biomedical Research Center Qatar University Doha Qatar; ^2^ Department of Mechanical and Industrial Engineering, College of Engineering Qatar University Doha Qatar; ^3^ Department of Mechanical Engineering TOBB University of Economics and Technology Ankara Turkey; ^4^ Department of Surgery, Trauma and Vascular Surgery, Hamad General Hospital, Hamad Medical Corporation Doha Qatar; ^5^ Clinical Medicine Weill Cornell Medicine Doha Qatar; ^6^ Department of Mechanical Engineering Middle East Technical University Ankara Turkey; ^7^ Department of Electrical Engineering, College of Engineering Qatar University Doha Qatar; ^8^ Department of Biomedical Science, College of Health Sciences Qatar University Doha Qatar

**Keywords:** abdominal aortic aneurysm, computational fluid dynamics, hemodynamics, rupture, wall shear stress

## Abstract

Abdominal aortic aneurysms (AAAs) are a serious medical condition that may culminate in internal bleeding and death. Clinicians are expected to assess the rupture risk of AAAs accurately to determine the mode and timing of intervention. In general practice, AAA diameter and growth rate are used as rupture risk indicators. However, numerous cases have been reported where relying solely on these two AAA characteristics has proven insufficient, suggesting that other biomechanical factors deserve further consideration. This paper aims to investigate the involvement of disturbed hemodynamics in AAA rupture. Twenty‐two AAA cases that had progressed to the point where surgical intervention was necessitated were assessed to examine the flow dynamics around the rupture sites. Using computational fluid dynamics (CFD), four key wall shear stress (WSS)‐related hemodynamic parameters were calculated for each studied case, namely the time‐averaged wall shear stress (TAWSS), oscillatory shear index (OSI), endothelial cell activation potential (ECAP), and relative residence time (RRT). CFD geometries were developed exclusively using patient computed tomography images, and simulations were run with general physiological boundary conditions to demonstrate a clinically practical, low‐input CFD pipeline. The study found that analyzing the spatial distribution of the WSS‐related hemodynamic parameters can be a powerful approach for predicting the site of rupture in AAAs. Low TAWSS and high OSI/ECAP/RRT regions (specifically within the ranges: TAWSS 0–0.5 Pa, OSI 0.35–0.5, ECAP 1.6–2.0 Pa^−1^, RRT 24–30) were found to be high‐risk locations for rupture. Additionally, the simultaneous analysis of all four parameters was critical for rupture risk assessment.

## Graphical abstract

Abdominal aortic aneurysms (AAAs) pose a substantial threat to public health due to their risk of rupture, which can be life‐threatening. Current clinical practice relies primarily on AAA diameter and growth rate as indicators to determine if surgical treatment is imperative. This study investigates the integration of WSS‐related hemodynamic parameters, from CFD analysis, into rupture risk assessment procedures, as this can greatly assist clinicians in more accurately predicting rupture risk for AAAs and the subsequent need for surgical intervention.

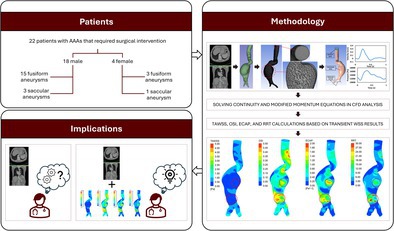

## 1 Introduction

An aortic aneurysm is a serious condition in which the aorta is abnormally enlarged in a balloon‐like bulge. The majority of aortic aneurysms are on the abdominal side, and these are known as abdominal aortic aneurysms (AAAs). The prevalence of AAAs in patients above the age of 50 is reported as 0.5%–1% in women and 4%–8% in men [1, 2]. The normal diameter of the abdominal aorta is reported to be 2–2.5 cm [3]. When the aortic dilation surpasses 50% of the normal abdominal aorta diameter, this condition is referred to as an AAA [4]. Unfortunately, AAA has a silent progression, and most of the patients with AAAs do not exhibit any distinctive clinical symptoms during the development of the disease [5]. AAA is often detected by coincidence during the diagnosis process of another disease or a general health screening [6]. The worst‐case scenario of an AAA is the rupture of the aneurysm, in which 80% of occurrences result in death [7]. Therefore, surgical intervention may be required to prevent an impending AAA rupture. Deciding on the cases requiring surgical operation is a challenging task for clinicians [3]. In today's clinical practice, surgery is advised if the AAA diameter exceeds 5.5 cm or the AAA expansion rate is greater than 1 cm/year [8, 9]. It is reported that there are non‐ruptured AAAs with a 9 cm diameter [10] and ruptured AAAs with diameters smaller than 5.5 cm [11, 12, 13, 14, 15]. Consequently, a comprehensive rupture risk assessment is crucial for more accurately predicting the risk of rupture. For this purpose, demystifying the biomechanical environment in AAAs would help determine potential treatment strategies and understand the etiology of the disease [3]. Clinical tools such as Doppler ultrasonography can only provide the blood flow velocities at focused cross‐sectional areas and cannot visualize the three‐dimensional (3D) flow in detail [16, 17, 18]. Therefore, computational engineering methods are required to be applied to obtain the detailed flow parameters [19, 20, 21]. Computational fluid dynamics (CFD) simulations are among the most powerful methods to reveal the complex flow field in the aneurysm [22, 23, 24]. In this method, the flow geometry is first generated using three‐dimensional imaging tools, and clinically measured boundary conditions are applied to simulate the flow in a virtual environment [25]. Here, the physically governing flow equations are solved in discretized spatial and time domains [26]. This way, a realistic 3D blood flow is created to determine the critical flow parameters at any point in the investigated geometry.

The critical hemodynamic parameters can be summarized as the blood flow velocity, fluid pressure, wall shear stress (WSS), oscillatory shear index (OSI), endothelial cell activation potential (ECAP), and relative residence time (RRT) [3, 27]. We have recently reviewed the literature on the existing knowledge relating disturbed hemodynamics parameters to AAA rupture. In the same paper, we demonstrated the practical calculation of these parameters. We encourage interested readers to see this paper [28]. These flow‐dependent hemodynamic parameters are useful for characterizing the disturbed flow inside AAAs, which can play an extremely important role in discerning the progression and rupture mechanisms of AAAs [29, 30, 31], since the rupture is essentially the mechanical failure of the arterial structure under the effect of hemodynamic forces. The disturbed hemodynamics in AAAs deteriorate the proper remodeling process of the artery. Consequently, altered hemodynamics in AAAs lead to a mechanically degenerated arterial structure, which has been shown to increase the risk of rupture [32]. In the literature, numerous computational investigations have been performed, aiming to determine the most effective hemodynamic parameters for predicting AAA rupture [33, 34, 35, 36, 37]. It has been reported that the flow velocities in the aneurysm sac decrease depending on the aneurysmal enlargement, which reduces the level of WSS on the AAA wall [3]. Recent investigations on the relevance of disturbed hemodynamics with AAA rupture mechanics provided insight into the potential of CFD as a useful predictor of AAA rupture risk [38, 39]. Recirculating flows form in the enlarged region, which also increases the residence time of the red blood cells around the AAA wall, thereby increasing the possibility of intraluminal thrombus (ILT) formation [40]. According to several studies, the biomechanical environment in the AAA sac is severely influenced by the patient‐specific AAA geometry and ILT formation [41, 42]. Therefore, modeling the ILT on the AAA wall is necessary for obtaining accurate results. Because of the highly complex geometries of patient‐specific AAA models, local variations are commonly observed in the values of the hemodynamic parameters [43]. Therefore, each patient needs to be investigated individually to conduct a reliable hemodynamic assessment [3]. A number of existing studies have concentrated on the WSS‐related parameters by elucidating the magnitudes and directions of the shear forces on the AAA wall and concluded that the regions with low WSS and high OSI are more prone to rupture [31, 40, 44, 45]. While useful, these studies have included only a few ruptured/unruptured cases; hence, comprehensive investigations could not be conducted. This is mainly because of the challenging tasks of pre‐processing and post‐processing steps of CFD models, limiting the number of CFD models to be studied in a feasible time.

It is essential to note that in real‐world clinical practice for the diagnosis and treatment of AAAs, the imaging modality of choice is usually contrast‐enhanced computed tomography angiography (CTA) [46]. This method is specifically preferred as it is a fast, safe, and relatively non‐invasive way to obtain detailed information about the anatomy of AAAs and the surrounding tissue [47]. Nevertheless, standard CTA systems are limited to the imaging of anatomical details and are not equipped to provide the parameters necessary for the investigation of hemodynamic changes in the AAA region, such as the corresponding velocity and pressure of blood [48]. Although in routine patient care, the blood pressure (BP) is commonly measured using a cuff BP gauge, the remoteness of the location of this measurement from the AAA itself means that it cannot be reliably considered as the blood pressure within the aorta [49]. Consequently, to incorporate CFD into clinical practice, while minimizing the additional resources and time required, a simplified CFD workflow using only CT‐derived geometrical information and standard boundary conditions would be the most straightforward approach. In addition, although CT scans provide considerable information about the morphology of arterial walls, the mechanical properties of these walls cannot be measured by standard CTA machines and may vary considerably from patient to patient [50]. Therefore, the most practical approach, with the available CTA data, would be to exclude the arterial wall and only model the aortic lumen for simulations.

In the current investigation, a comprehensive computational AAA analysis was conducted, with 22 selected AAA cases using a streamlined CFD workflow based exclusively on the geometrical information acquired from CT scans, and incorporating standard boundary conditions from the literature [51]. Furthermore, while the majority of previous AAA computational studies have focused mainly on the time‐averaged wall shear stress (TAWSS) and OSI parameters, here the healthy and enlarged abdominal aortas were compared and analyzed in terms of four hemodynamic parameters: TAWSS, OSI, ECAP, and RRT. Consideration was also given to the effect of the degree of ILT on the values of these parameters. Additionally, retrospective cases with ruptured AAAs made up the majority of the evaluated cases in the study, which allowed for the assessment of the correlation between the spatial distribution of WSS parameters and actual ruptured sites on the AAAs. The latter analysis was performed blindly. Therefore, the site of rupture was not known during the assessment of the computational modeling results.

## 2 Methods

### 2.1 Model Geometries

Computed tomography (CT) images of 1 healthy aorta and 22 AAA cases were obtained from Hamad General Hospital, Department of Surgery, Trauma and Vascular Surgery Center, under ethical approval MRC‐02‐20‐134. The study population comprises 18 male patients and 4 female patients with AAAs, all of whom had reached a stage in the condition where surgical intervention had been necessitated. The average ages were 65 and 66 for male and female patients, respectively. The youngest patient in the male group was 31 years old, and the youngest female was 43. Patients' data were divided into two groups: the low ILT group for AAA cases with a small ILT deposition or with no ILT deposition, and the high ILT group for AAA cases with a severe rate of ILT deposition. ILT deposition case classification was carried out according to clinicians' reports, which were prepared based on the patient's CT images. Although ILT was not explicitly modeled, given that the considered geometry corresponded to the aorta lumen, this geometry inherently represents the AAA flow domain in the presence of ILT.

**FIGURE 1:**
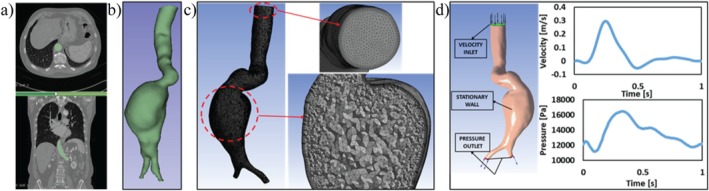
CFD model generation: (a) Patient‐specific CT images. (b) 3D geometry segmentation by using 3D Slicer software. (c) Generated a tetrahedral mesh with inflation layers on the boundaries. (d) Transient CFD analysis boundary condition locations on the AAA model, and Velocity–Time and Pressure–Time graphs used as inlet and outlet conditions [28] (Reprinted with permission).

Figure 1 summarizes the steps for CFD model generation. The obtained CT images, an example of which can be seen in Figure 1a, were segmented using the open‐source 3D Slicer (https://www.slicer.org) software, with validation by the clinicians involved in the study. After that, AAA geometries in “.stl” format were re‐meshed and re‐constructed using the Mesh Mixer (http://www.meshmixer.com/Autodesk Inc., San Rafael, California, USA) software. Finally, the patient geometries in the “.stl” format were converted to solid geometries for obtaining the blood flow zones suitable for CFD analysis, using SpaceClaim (ANSYS Inc., Canonsburg, Pennsylvania, USA) software. The isolated AAA geometry for each patient extended from the beginning of the descending aorta to the two common iliac arteries, as shown in Figure 1b. In each geometry, there is one inlet zone and two outlet zones. Such geometries were shown to result in realistic hemodynamic findings using CFD simulations [31, 36, 40, 41, 42, 52, 53, 54, 55].

### 2.2 Mesh Generation and Sensitivity Analysis

The ANSYS default mesh generator (ANSYS Inc., Canonsburg, PA, USA) was used to generate tetrahedral meshes of the models, with hexahedral inflation layers as shown in Figure 1c. The appropriate *y* + value was established as *y* + < 2, which is ideal for the viscous sublayer and the SST turbulence model to calculate the shear stresses close to the wall accurately, as explained by Bredberg et al. [56]. According to the *y* + calculations, the first layer height, number of layers, and growth rate were chosen to be 0.1 mm, 1.0, and 1.1, respectively. To ensure the mesh utilized was of sufficiently high quality, mesh generation was conducted in such a way that all mesh elements had a maximum skewness of less than 0.95, an aspect ratio less than 100, and a minimum orthogonal quality higher than 0.1, for each simulation. The average number of mesh elements used in all cases was close to one million.

An essential part of using CFD to investigate vascular hemodynamics is carrying out a mesh sensitivity analysis to verify that the numerical uncertainty associated with spatial discretization is acceptable. Of the approaches available for this, the use of the grid convergence index (GCI), originally proposed by Roache, is a rigorous and widely adopted method [57, 58]. To accurately assess convergence using this method, it is important to select a variable that is representative of the parameters to be investigated. Therefore, the mean TAWSS was employed for GCI calculations in this study. This variable was determined for the third cardiac cycle in each case, to eliminate the transient effects of the two previous cycles. From the 22‐patient cohort considered, these calculations were carried out for three patient geometries. Patients 3, 12, and 4 were selected as examples of a typical fusiform AAA morphology, a typical saccular AAA morphology, and the most complex geometry considered, respectively. For each patient in question, the mean TAWSS results from transient simulations were compared for fine, medium, and coarse meshes, where the medium mesh results correspond to those from the final selected mesh size, in accordance with GCI calculation protocols. All of these simulations were carried out using identical solver settings and a time step size of 0.01 s. The important quantities when calculating the GCI include the refinement ratios, $r_{21}$ and $r_{32}$, which can be calculated according to Equations (1) and (2) below.

(1) $$r_{21}={\left(\frac{N_{1}}{N_{2}}\right)}^{1/3}$$

(2) $$r_{32}={\left(\frac{N_{2}}{N_{3}}\right)}^{1/3}$$

In the equations, $N_{1}$, $N_{2}$, and $N_{3}$ are the number of mesh elements for the fine, medium, and coarse meshes, respectively, for each patient. The observed order of convergence also needs to be determined, using Equations (3) and (4).

(3) $$p=\frac{1}{\ln\left(r_{21}\right)}\left|\ln\;\left(\left|\frac{\phi_{3}-\phi_{2}}{\phi_{2}-\phi_{1}}\right|\right)+\ln\;\left(\frac{r_{21}^{p}-s}{r_{32}^{p}-s}\right)\right|$$

(4) $$s=\mathrm{sign}\;\left(\frac{\phi_{3}-\phi_{2}}{\phi_{2}-\phi_{1}}\right)$$

In these equations, $\phi_{1}$, $\phi_{2}$ and $\phi_{3}$ represent the mean TAWSS for the fine, medium, and coarse meshes, respectively, for each patient. On the other hand, the sign function will return the sign of the argument in brackets (it can have a value of −1, +1, or 0). With the aforementioned quantities in hand, the GCI values can then be calculated, according to Equations (5) and (6).

(5) $${\mathrm{GCI}}_{21}=F_{s}\cdot \frac{|\phi_{1}-\phi_{2}|}{\phi_{1}\left(r_{21}^{p}-1\right)}\times 100\%$$

(6) $${\mathrm{GCI}}_{32}=F_{s}\cdot \frac{|\phi_{2}-\phi_{3}|}{\phi_{2}\left(r_{32}^{p}-1\right)}\times 100\%$$

here ${\mathrm{GCI}}_{21}$ is for comparing the medium mesh with the fine mesh, and ${\mathrm{GCI}}_{32}$ is for contrasting the coarse mesh with the medium mesh. Meanwhile, $F_{s}$ represents the safety factor used in the original formula. In accordance with the procedure defined by Roache, a safety factor of 1.25 can be used when comparing the solution for a selected quantity over three grid sizes, provided that the solutions are in the asymptotic range (AR). For this condition to be satisfied, AR, defined in Equation (7) below needs to have a value close to 1 [59].

(7) $$\mathrm{AR}=\frac{{\mathrm{GCI}}_{32}}{r_{21}^{p}\cdot {\mathrm{GCI}}_{21}}$$

From the GCI results in Table 1, it can be seen that the AR is very close to 1 for every patient analyzed, which shows that the refinement ratios are adequate and GCI can be reliably used. Additionally, it can be seen that the medium mesh exhibits acceptable spatial convergence across all three representative geometries, with values well below 5% in all cases [60]. In particular, the GCI values comparing the medium mesh with the fine mesh are below 2% in all cases, which indicates excellent spatial convergence. These results clearly demonstrate that the uncertainty associated with the spatial discretization of the medium mesh is acceptable, and this mesh resolution can therefore be reliably used for all patient simulations. Following this, time sensitivity analysis was evaluated using the medium mesh for Patient 4, the most complex modeled AAA geometry. For this, the total simulation time of 3 s remained unchanged, while simulations were run with three different time step sizes of 0.01, 0.005, and 0.0025 s, respectively. Once again, the mean TAWSS was selected as the quantity to be considered and calculated for the third cardiac cycle for each simulation. Here, simple percentage difference calculations were used to compare the mean TAWSS value for each time step with that of the time step half its size.

**TABLE 1:** Results for mesh sensitivity analysis based on GCI.

	GCI mesh level	No. of elements (millions)	Avg. TAWSS (Pa)	*R*	GCI (%)	AR
Patient 3	Fine	2.6893	0.48797	1.35	1.965	
	Medium	1.1046	0.48954	1.35	2.359	0.997
	Coarse	0.4501	0.49145	—	—	
Patient 4	Fine	3.9755	0.60314	1.34	0.037	
	Medium	1.6459	0.60392	1.34	0.198	0.999
	Coarse	0.6797	0.60815	—	—	
Patient 12	Fine	2.8772	0.85132	1.34	0.459	
	Medium	1.1984	0.85519	1.34	1.022	0.995
	Coarse	0.5018	0.86378	—	—	

From the percentage difference results in Table 2, it can be seen that the percentage difference relative to the next smallest time step in both cases was less than 0.2%. Therefore, temporal convergence for mean TAWSS was shown to be excellent for the evaluated time step sizes, and a time step of 0.01 s can be reliably used for all patients [61].

**TABLE 2:** Results for time sensitivity analysis based on percentage differences.

	Timestep size (s)	No. of elements (millions)	Avg. TAWSS (Pa)	% Difference
Patient 4	0.0100	1.6459	0.60392	0.172
	0.0050	1.6459	0.60288	0.084
	0.0025	1.6459	0.60339	—

### 2.3 Numerical Model

The blood was modeled as a non‐Newtonian fluid in the computational domain with a density of 1047 kg/m^3^ [62, 63, 64] and a molar mass of 64.458 kg/mol [65]. The blood viscosity was modeled using the Bird‐Carreau non‐Newtonian model, and the high and low shear viscosity levels were found to be 0.0035 Pa s and 0.056 Pa s, respectively [66]. When Reynolds numbers were calculated for each patient, it was observed that the Reynolds numbers ranged from 2000 to 3000. Even though the estimated Reynolds numbers are in the transition zone, the turbulent model is utilized to better capture the shear stresses near the wall.

The blood was assumed to be a homogeneous and incompressible fluid. The physically governing equations, namely continuity (Equation 8), modified momentum (Equation 9), and turbulence model equations, are solved in the numerical model.

(8) $$\frac{\partial \rho }{\partial t}+\frac{\partial }{\partial x_{j}}\left(\rho u_{j}\right)=0$$

(9) $$\frac{\partial }{\partial t}\left(\rho u_{i}\right)+\frac{\partial }{\partial x_{j}}\left(\rho u_{i}u_{j}\right)=\frac{\partial P^{\prime }}{\partial x_{i}}+\frac{\partial }{\partial x_{j}}\;\left[\mu_{\mathrm{eff}}\left(\frac{\partial u_{i}}{\partial x_{j}}\right.+\left.\frac{\partial u_{i}}{\partial x_{i}}\right)\right]+S_{M}$$

In the equations, ρ indicates the density, u indicates the velocity vector, and the letters $P^{\prime }$, $\mu_{\mathrm{eff}}$, and $S_{M}$ stand for the modified pressure, effective viscosity accounting for turbulence, and the sum of body forces, respectively.

The turbulence model used is the shear stress transport (SST) model, which operates by solving a turbulence/frequency‐based model (k–ω) at the wall and k‐ε in the bulk flow [67, 68]. A mixing function makes sure that switching between the two models is seamless. By utilizing this approach, the model can accurately forecast the separation, which the k‐ε model alone was unable to achieve. Similar to the zero‐equation model, the k‐ε model is built on the idea of eddy viscosity, which enables Equation (10).

(10) $$\mu_{\mathrm{eff}}=\mu +\mu_{t}$$

In Equation (10), $\mu_{t}$ is the turbulence viscosity, and the k‐ε model presupposes the relationship between turbulence viscosity, turbulence kinetic energy, and dissipation. In the definition of $\mu_{t},$ a constant $C_{\mu }$ is used as given in Equation (11).

(11) $$\mu_{t}=C_{\mu }\rho \frac{k^{2}}{\varepsilon }$$

Another turbulence model that is required for the application of the SST model is the k‐ω equation. The k‐ω model does not require the intricate nonlinear damping functions needed for the k‐ε model, which makes it more precise and durable. According to the k‐ω models, the relationship between the turbulent viscosity, turbulence kinetic energy, and turbulence frequency can be defined as given in Equation (12).

(12) $$\mu_{t}=\rho \frac{k}{\omega }$$

### 2.4 Boundary Conditions

Transient fluid flow simulations were carried out using ANSYS CFX (ANSYS Inc., Canonsburg, Pennsylvania, USA) with a 0.01 s time step for 3 s, equal to three cardiac cycles. In this study, three cardiac cycles were simulated for the computational model, and the results of the third cycle were considered in the numerical analysis to eliminate the transient effects in the first two cycles. Previous studies showed that, as the blood flow exiting the heart enters the aorta, the flow in the aorta is not in a fully developed form, and it is not distributed symmetrically [3, 69, 70, 71, 72]. Therefore, in the analysis, the input and output boundary conditions were defined as normal to the surface boundary, as shown in Figure 1d, instead of using a fully developed flow profile in order to ensure physiological consistency. The inlet is defined using a velocity boundary condition, whereas the outlets are modeled using an open boundary condition at the two common iliac arteries, in order to account for potential flow reversal. As has been previously mentioned, based on the fact that CTA is used as the standard imaging modality when diagnosing and treating AAAs, the velocity and pressure for each individual patient at the region of concern would typically not be available. Hence, in all the models, a common velocity and pressure flow profile from previous literature has been used [51]. The velocity and pressure graphs for these profiles are shown in Figure 1d. The boundary conditions were established using expressions that accurately replicated these established physiological velocity and pressure profiles. For both the inlet and outlet boundaries, a medium turbulence intensity of 5% was selected.

### 2.5 WSS‐Related Parameters

WSS‐related parameters were used to identify the critical locations that are more prone to rupture. The employed WSS‐related parameters are the TAWSS, OSI, ECAP, and RRT. For each patient, the values of WSS‐related parameters are determined using the third cycle of the CFD analysis to avoid the transient effects in the first two cardiac cycles. The WSS components (WSSx, WSSy, and WSSz) in x, y, and z directions were exported using the CFD Post, and the WSS‐related parameters were calculated using MATLAB software (MathWorks, Natick, Massachusetts, USA). Following this, the calculated parameters were imported into CFD Post to view the corresponding contour plots.

Equation (13) defines TAWSS. The absolute values of WSS components in the x, y, and z directions are used for the duration of T, which is the time length of one full cardiac cycle. In Equation (13), $\tau_{w}$ denotes the instantaneous WSS vector, which is determined by the CFD simulations.

(13) $$\text{TAWSS}=\frac{1}{T}\;\left(\int_{0}^{T}\left|\tau_{w}\right|\;\mathrm{dt}\right)$$

OSI is an important hemodynamic parameter that explains the directional variation of WSS from the prevailing axial direction. OSI can be defined in various forms as shown in Equation (14). OSI values vary from 0 to 0.5, with a value of 0.5 denoting WSS with a zero temporal average and a value of 0 denoting unidirectional WSS [25, 26, 73].

(14) $$\mathrm{OSI}=\frac{1}{2}\left(1-\frac{\left|\int_{0}^{T}\tau_{w}\;\mathrm{dt}\right|}{\int_{O}^{T}\left|\tau_{w}\right|\mathrm{dt}}\right)=\frac{1}{2}\left(1-\frac{\frac{1}{T}\left|\int_{0}^{T}\tau_{w}\;\mathrm{dt}\right|}{\frac{1}{T}\int_{O}^{T}\left|\tau_{w}\right|\mathrm{dt}}\right)=\frac{1}{2}\left(1-\frac{\frac{1}{T}\left|\int_{0}^{T}\tau_{w}\;\mathrm{dt}\right|}{\text{TAWSS}}\right)$$

ECAP is used as a measure to define the thrombogenic susceptibility of the AAA wall and is calculated by finding the ratio of OSI and TAWSS as provided in Equation (15) [74]. High OSI and low TAWSS conditions increase the probability of thrombus formation on the artery walls.

(15) $$\text{ECAP}=\frac{\mathrm{OSI}}{\text{TAWSS}}$$

RRT is another hemodynamic parameter that is used to assess the state of the degraded flow. The definition of RRT is provided in Equation (16) [75]. It should be noted that RRT is inversely proportional to the numerator of OSI, as shown in Equation (17). The detailed calculation methods for TAWSS, OSI, ECAP, and RRT are also explained in [28].

(16) $$\mathrm{RRT}=\frac{1}{\left(1-2\times \mathrm{OSI}\right)\times \text{TAWSS}}$$

(17) $$\mathrm{RRT}=\frac{1}{\frac{1}{T}\left|\int_{0}^{T}\tau_{w}\;\mathrm{dt}\right|}$$

## 3 Results

The CT images were reviewed and analyzed by a radiologist to identify the rupture locations independent of the surgeons' intraoperative findings. Initially, CFD analysis was performed blindly at Qatar University to pinpoint potential rupture locations on the AAAs without prior knowledge of the findings of radiologists and surgeons. However, for a more detailed analysis of the WSS‐related hemodynamic parameters, the known rupture locations were taken into account. These rupture locations are indicated in AAA contour plots in the paper with red circles. The results for CFD analysis for each patient, that is, the determined TAWSS, OSI, ECAP, and RRT ranges, were taken from within these red circles that encompass the rupture locations. Table 3 summarizes the CFD analysis results for all studied cases, along with patient demographics, comorbidities, and clinical AAA assessment.

**TABLE 3:** Summary of demographics, comorbidities, AAA size, and dynamic parameters (TAWSS, OSI, ECAP, and RRT) distribution.

Patient number	Age	Gender	HTN	DYSLIP	DM	CAD	Vasculitis	Smoking	Size in the last CT (cm)	TAWSS (Pa)		OSI		ECAP (Pa^−1^)				RRT					
										0–0.5	0.5–1.0	0.20–0.35	0.35–0.50	0.2–0.8	0.8–1.2	1.2–1.6	1.6–2.0	18–24	24–30	L‐ILT	H‐ILT	FS	SC
1	51	M	Yes	No	Yes	No	No	Yes	6.2	X			X				X		X	X		X	
2	62	M	Yes	Yes	Yes	No	No	Yes	7	X			X			X			X	X			X
3	61	M	Yes	Yes	Yes	No	No	Yes	9.3	X			X				X		X	X		X	
4	31	M	Yes	No	No	No	Yes	Yes	6	X			X				X		X	X		X	
5	64	M	Yes	Yes	No	No	No	No	8.5	X		X					X	X		X		X	
6	74	M	Yes	No	No	Yes	No	Yes	6.2	X			X				X	X		X		X	
7	54	M	Yes	Yes	Yes	Yes	No	Yes	4.5	X			X		X				X	X		X	
8	79	M	Yes	Yes	No	No	No	Yes	10	X			X		X			X		X		X	
9	73	M	Yes	Yes	Yes	No	No	No	5.1		X		X	X					X	X		X	
10	76	F	Yes	No	No	No	No	No	6.1	X			X				X		X	X		X	
11	80	M	No	Yes	No	No	No	Yes	9.9	X			X				X		X	X		X	
12	43	F	Yes	No	Yes	No	No	No	5.3	X		X					X		X	X			X
13	81	M	Yes	Yes	Yes	No	No	Yes	5.3	X			X				X		X		X	X	
14	77	F	No	No	No	Yes	Yes	No	5.9	X			X				X		X		X	X	
15	63	M	Yes	Yes	Yes	No	No	Yes	7	X			X				X		X		X	X	
16	66	M	No	Yes	Yes	No	No	Yes	9.5	X			X				X		X		X	X	
17	93	M	Yes	Yes	Yes	Yes	No	Yes	4.5	X			X				X		X		X		X
18	39	M	Yes	Yes	Yes	Yes	No	Yes	9.8	X			X			X			X		X	X	
19	80	M	Yes	Yes	Yes	Yes	No	Yes	7.3		X		X	X					X		X	X	
20	68	F	Yes	Yes	Yes	Yes	No	No	5.5	X			X	X				X			X	X	
21	72	M	Yes	Yes	Yes	Yes	No	No	5.7	X			X	X				X			X	X	
22	41	M	Yes	Yes	No	No	Yes	Yes	3.3	X			X				X		X		X		X

Abbreviations: CAD = coronary artery disease; DM = diabetes mellitus; DYSLIP = dyslipidemia; FS = fusiforme; H‐ILT = high intraluminal thrombus; HTN = hypertension; L‐ILT = low intraluminal thrombus; SC = saccular.

### 3.1 Patient Cohort Overview

**FIGURE 2:**
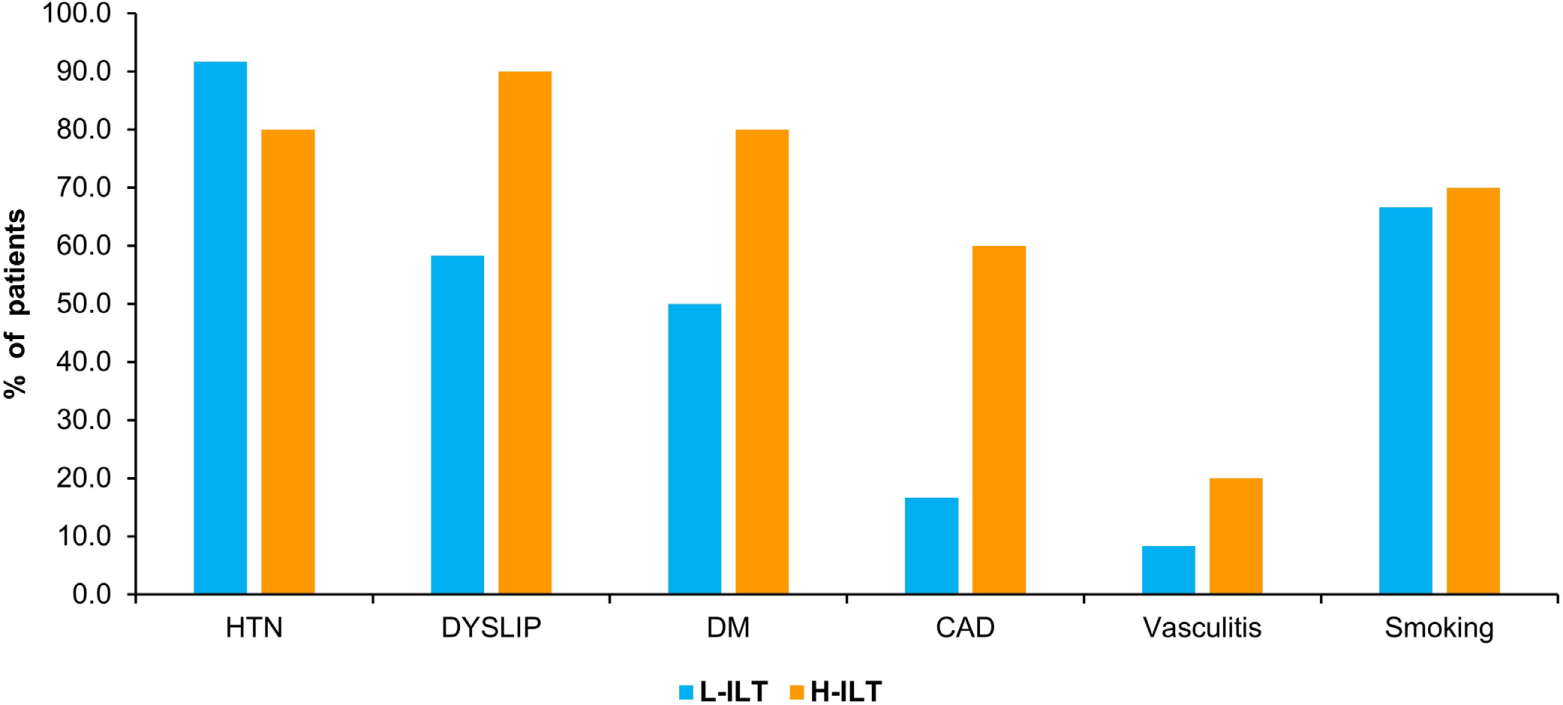
% of patients with comorbidities in no/low ILT group versus high ILT group.

A total of 22 cases of AAA were diagnosed; 19 had a ruptured aneurysm, and 3 had an impending rupture. All cases underwent surgical interventions. The mean age of patients was 64.9 ± 33.9 years. The study included 18 males (15 ruptured AAA cases and 3 impending ruptures) and 4 females (4 ruptured AAA cases). The main risk factors included hypertension (86.4%), dyslipidemia (72.7%), diabetes mellitus (63.6%), coronary artery disease (36.4%), vasculitis (13.6%), and if the patients are smokers (68.2%). The AAA geometries were grouped as no/low ILT or high ILT for further examination. There were 12 patients in the no/low ILT group (designated with L‐ILT in Table 3, topmost rows) and 10 patients in the high ILT group (designated with H‐ILT in Table 3, bottommost rows). CT images were also used to identify the level of ILT for each case. When comparing the percentage of patients with each of the respective comorbidities for the no/low ILT group with the high ILT group, it can be seen that these values are generally noticeably higher for the high ILT group. The percentage of patients with hypertension is the single exception to this trend, as there were 91.7% of patients with this comorbidity in the no/low ILT group versus 80% of patients in the high ILT group. For the patients who were smokers in both groups, there was less than a 4% difference, with the no/low ILT group having 66.7% of patients who were smokers versus 70.0% for the high ILT group. Nevertheless, the high ILT group still had a larger percentage. For all the other aforementioned comorbidities, there were significantly larger percentages of patients with these coexisting conditions in the high ILT group. Most noteworthy was the fact that 60.0% of the patients in the high ILT group had coronary artery disease, as compared to only 16.7% for the no/low ILT group. These trends can be seen in Figure 2 below.

**FIGURE 3:**
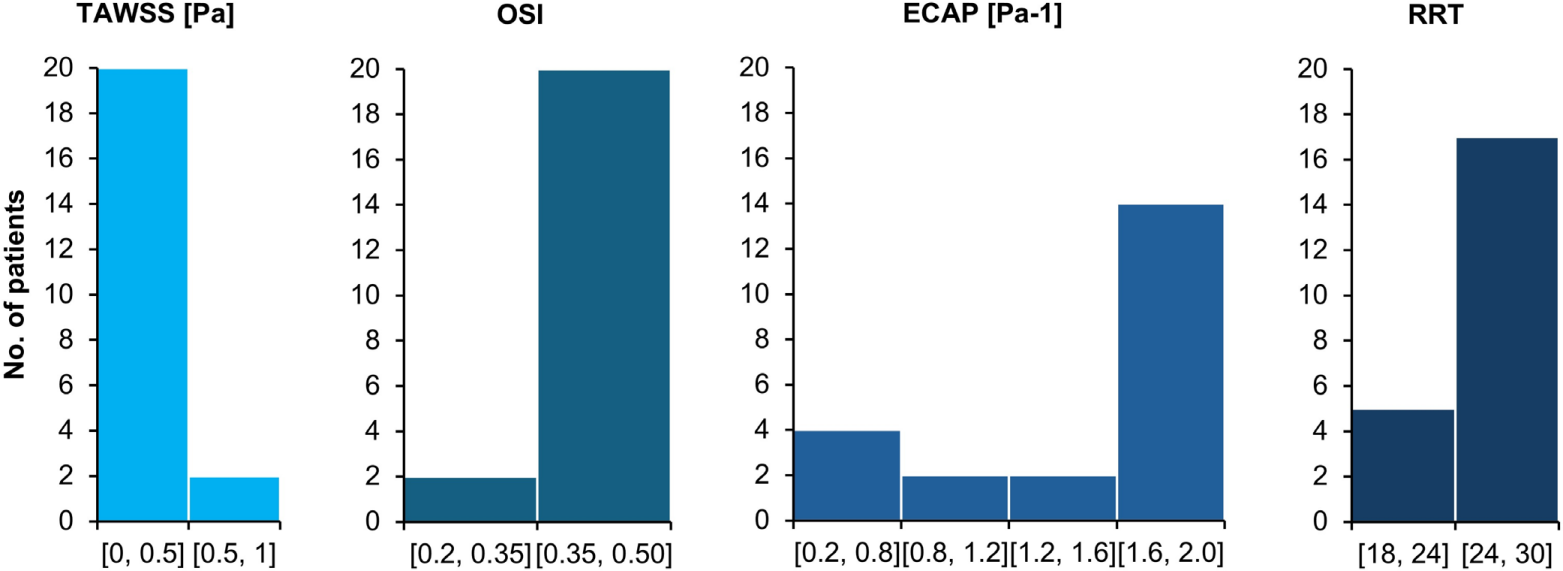
Histograms of TAWSS, OSI, ECAP, and RRT values for all the patients as a whole.

Also of particular note is the fact that in six of the ruptured AAA cases, the size of the aneurysm was ≤ 5.5 (31.6%). Concerning the value of the WSS‐related hemodynamic parameter values for all the patients as a whole, for the TAWSS values, 90.9% of cases were in the lower specified range (0–0.5 Pa), while 90.9% of patients had OSI values in the higher specified interval (0.35–0.50). Also, 63.6% had ECAP values in the highest specified range (1.6–2.0 Pa^−1^) regardless of the shape of the aneurysm. Finally, approximately 77.3% of patients had RRT values in the higher specified interval (24–30). This distribution of WSS‐related hemodynamic parameters can be seen in Figure 3 below.

### 3.2 CFD Results for Low ILT Cases

The CFD results were investigated in three groups: the healthy aorta, low ILT AAAs, and high ILT AAAs. Out of 22 cases, 12 were low ILT cases. The topmost rows in Table 3 present these cases (patients 1–12). Furthermore, the results were also categorized into two distinct subgroups: fusiform aneurysms and saccular aneurysms, since previous works demonstrated that geometrical differences in the aneurysm shape may be an important factor when investigating the rupture risk [31, 36, 37, 41, 76, 77].

**FIGURE 4:**
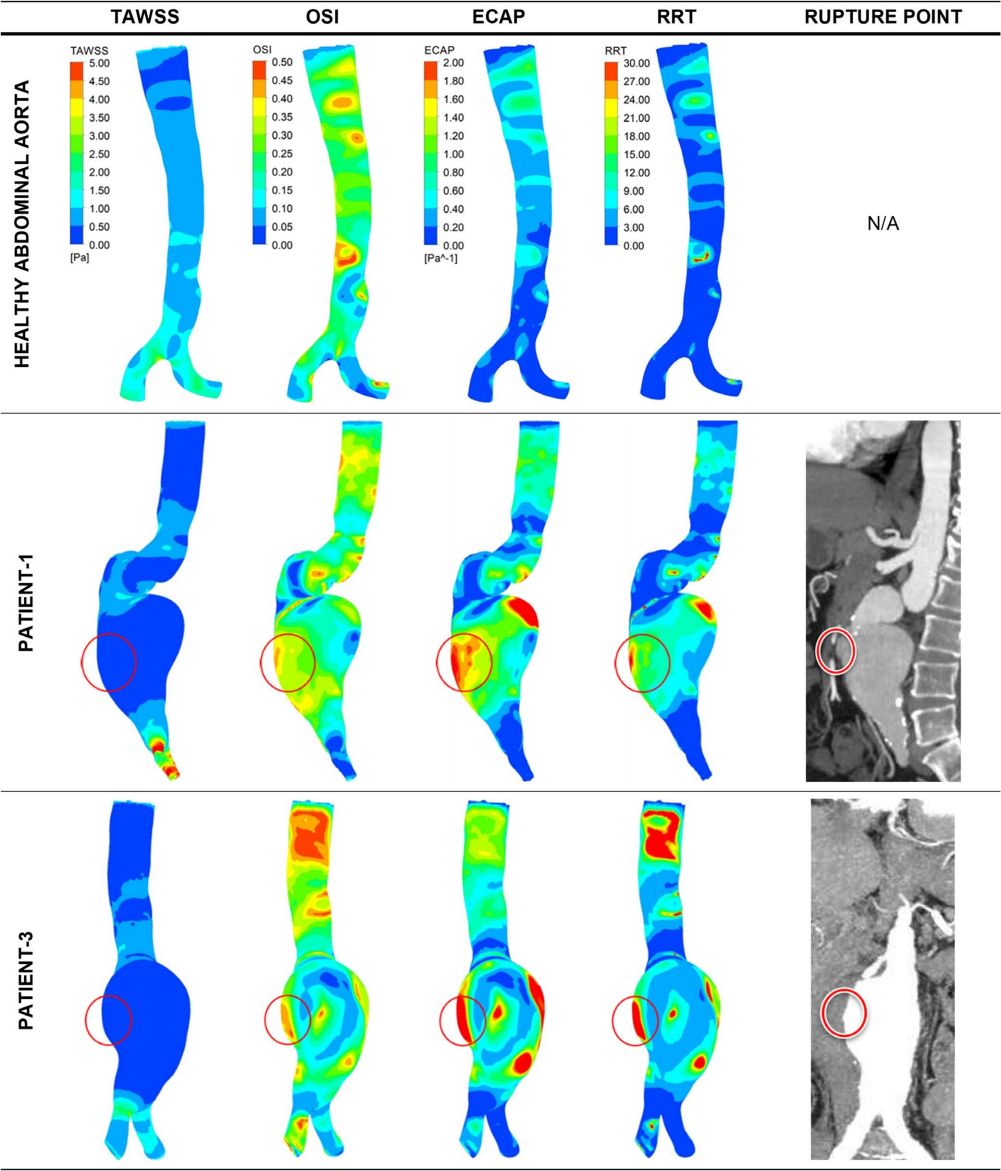
Distribution of WSS parameters of TAWSS, OSI, ECAP, and RRT in contour plots for healthy abdominal aorta and two representative ruptured fusiform low ILT aneurysm cases. The red circles indicate the clinician‐defined rupture site in the CT data and the contour plot area corresponding to the same location.

**FIGURE 5:**
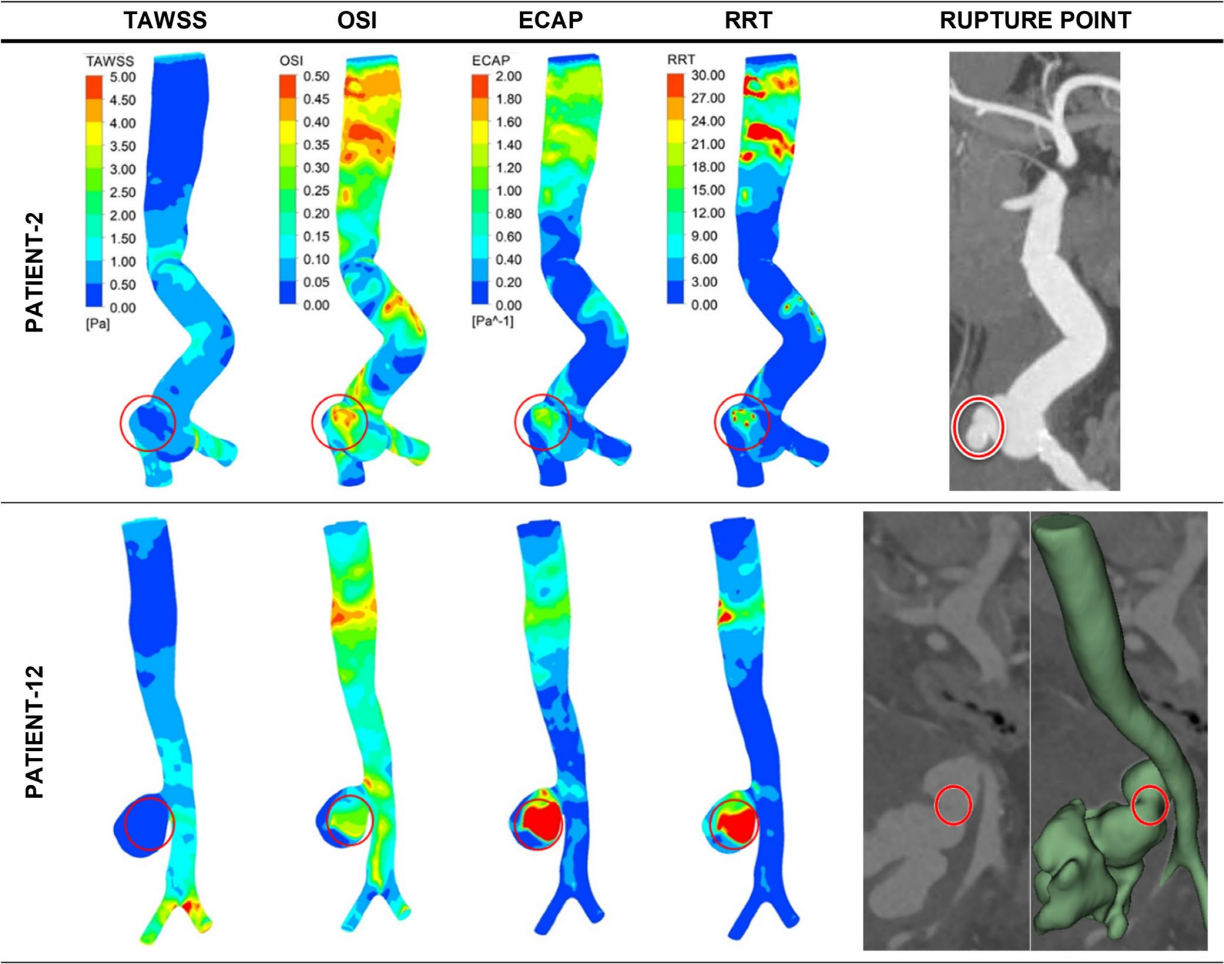
Distribution of WSS parameters of TAWSS, OSI, ECAP, and RRT in contour plots for ruptured saccular low ILT aneurysm cases. The red circles indicate the clinician‐defined rupture site in the CT data and the contour plot area corresponding to the same location.

**FIGURE 6:**
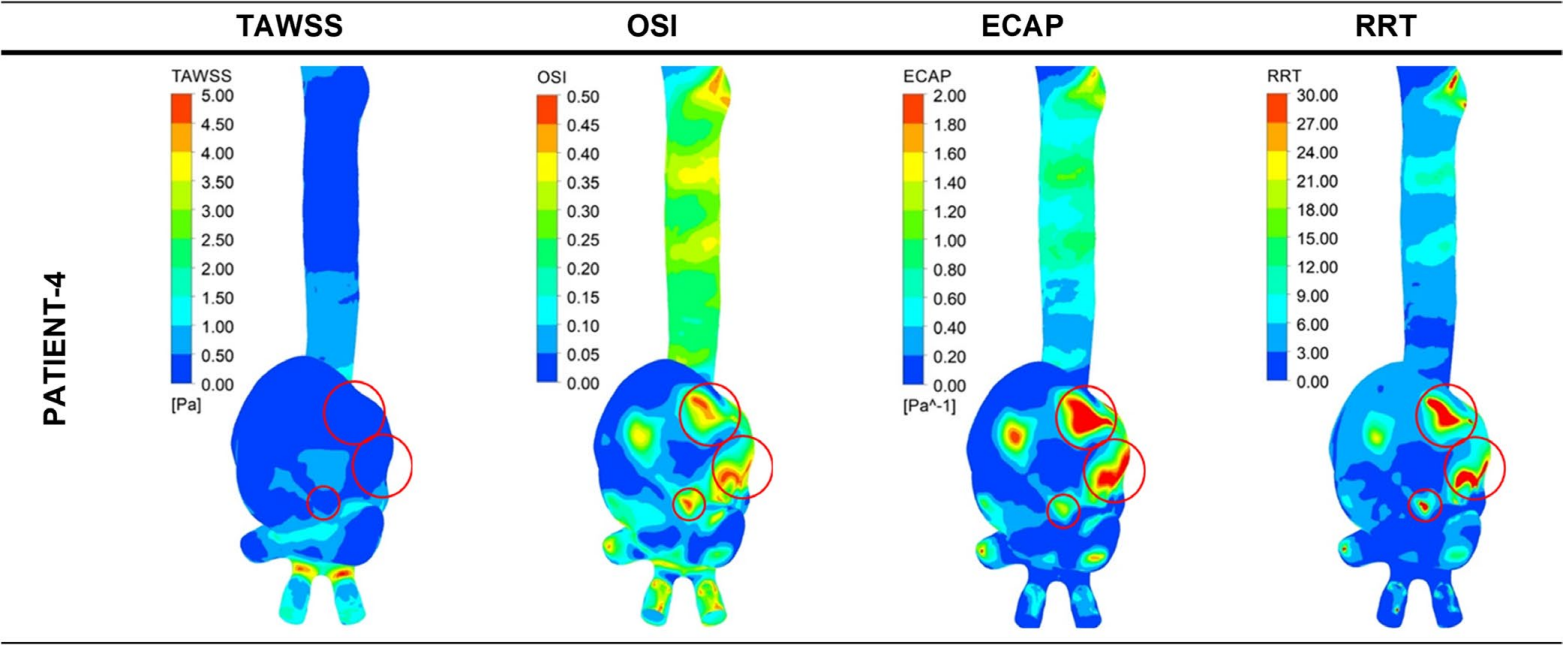
Distribution of WSS parameters of TAWSS, OSI, ECAP, and RRT in contour plots for impending rupture fusiform low ILT aneurysm cases. The red circles indicate the low TAWSS and high OSI, ECAP, and RRT areas.

Contour plots for the low ILT group are presented in Figures 4, 5, 6. Figure 4 presents two representative ruptured fusiform cases (patients 1 and 3) along with a healthy aorta case for comparison. The contour plots for all ruptured fusiform low ILT aneurysm cases (patients 1, 3, 5, 6, 7, 8, 9, 10, and 11) can be seen in Figure S1. Figure 5 presents ruptured saccular aneurysm cases (patients 2 and 12), and Figure 6 presents the impending rupture case. In Figure 4, for the healthy case at the lower section of the aorta, near the iliac arteries, where an AAA usually occurs, TAWSS values were between 0.5 and 1.5 Pa, OSI values reached as high as 0.5 in some small regions of the aorta, ECAP values were between 0 and 0.8 Pa^−1^, and RRT was less than 6 in general, but 30 in a single small region. It should be noted that for all patients besides the healthy case and the impending rupture cases, the results mentioned specifically refer to the observations made for the WSS‐related hemodynamic parameter values in close proximity to the rupture locations. For the impending rupture cases, the results are given in accordance with the predicted rupture locations.

**FIGURE 7:**
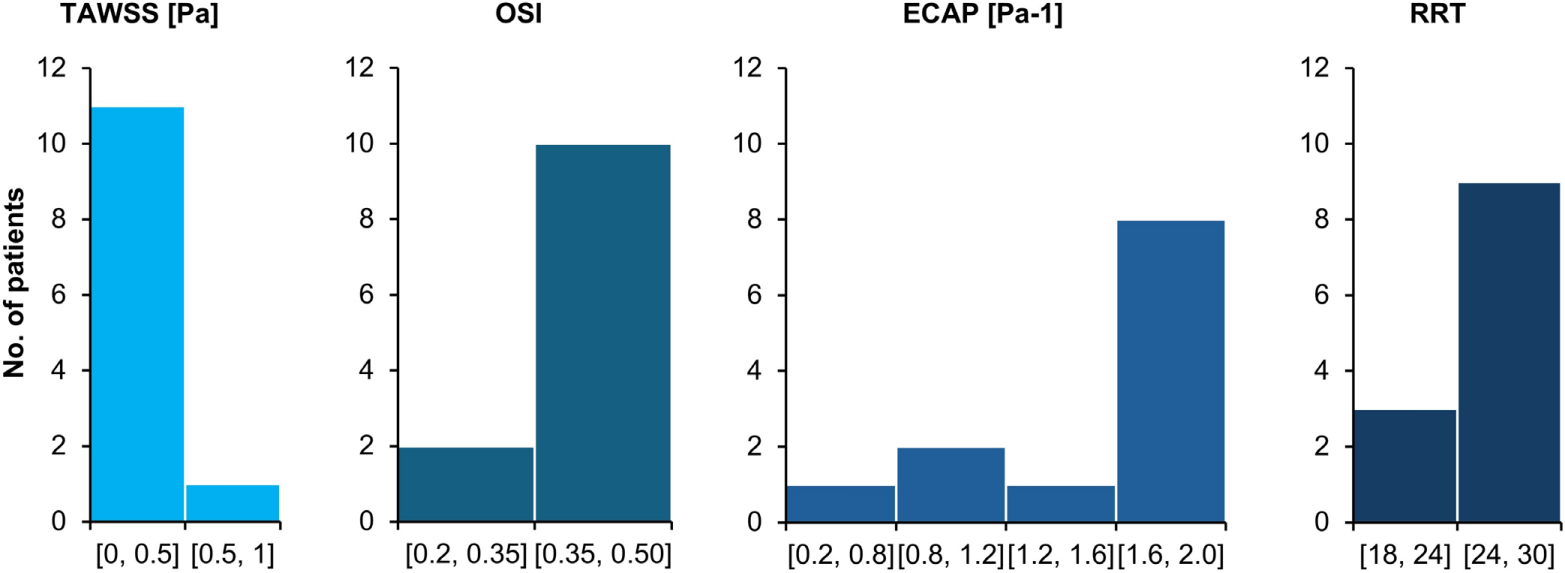
Histograms of TAWSS, OSI, ECAP, and RRT values for patients in the no/low ILT group.

For the aneurysm cases within the low ILT group as a whole, except for patient number 9, all patients had TAWSS values between 0 and 0.5 Pa; Patient 9 had TAWSS values between 0.5 and 1 Pa. Except for patients 5 and 12, all patients had OSI values between 0.35 and 0.50; patients 5 and 12 instead had OSI values between 0.20 and 0.35. With the exception of patients 2, 7, 8, and 9, all ECAP values were between 1.60 and 2.00 Pa^−1^; Patient 2 had ECAP values between 1.2 and 1.6 Pa^−1^, patients 7 and 8 had ECAP values between 0.8 and 1.2 Pa^−1^, and Patient 9 had ECAP values between 0.2 and 0.8 Pa^−1^. Except for patients 5,6, and 8, all patients in this group had RRT values between 24 and 30; patients 5, 6, and 8 had RRT values between 18 and 24. This apportionment of WSS‐related hemodynamic parameters can be more clearly seen in Figure 7 below.

The trend observed for the fusiform aneurysm cases was similar to that observed for all the aneurysm cases within the low ILT group, as 10 out of 12 patients in this group had fusiform aneurysms. When Figure 5 is analyzed, the two saccular aneurysm cases within the low ILT group had similar TAWSS and RRT values, with ranges of 0 to 0.5 Pa and 24 to 30, respectively. However, the OSI value was between 0.35 and 0.5 for Patient 2, and between 0.20 and 0.35 for Patient 12. In addition, the ECAP value was between 1.20 and 1.60 Pa^−1^ for Patient 2, and between 1.60 and 2.00 Pa^−1^ for Patient 12.

Even though the aneurysm of Patient 4 shown in Figure 6 was for an impending rupture, some regions had a combination of TAWSS values between 0 and 0.50 Pa, OSI values between 0.35 and 0.50, ECAP values between 1.60 and 2.00 Pa^−1^, and RRT values between 24 and 30, which were comparable to the values observed in the rupture locations for the ruptured aneurysm cases.

### 3.3 CFD Results for High ILT Cases

The AAA volume and flow channel shape may decrease due to high ILT accumulation. Therefore, the flow profile of the same AAA with or without ILT deposition can have a significant difference. As a result of high ILT accumulation, the flow channel in the AAA can take a more tubular shape.

**FIGURE 8:**
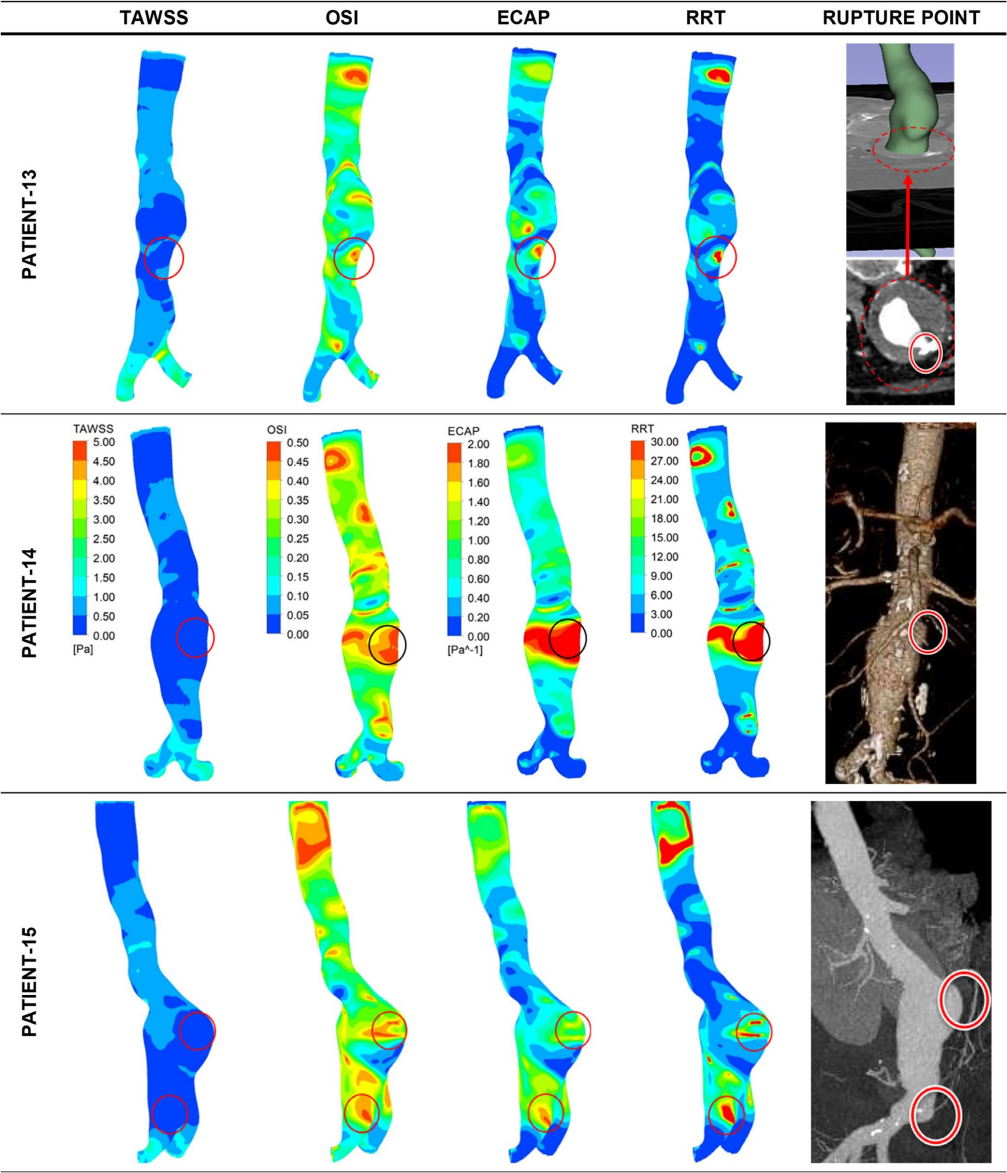
Distribution of WSS parameters of TAWSS, OSI, ECAP, and RRT in contour plots for three representative ruptured fusiform high ILT aneurysm cases. The red circles indicate the clinician‐defined rupture site in the CT data and the contour plot area corresponding to the same location.

**FIGURE 9:**
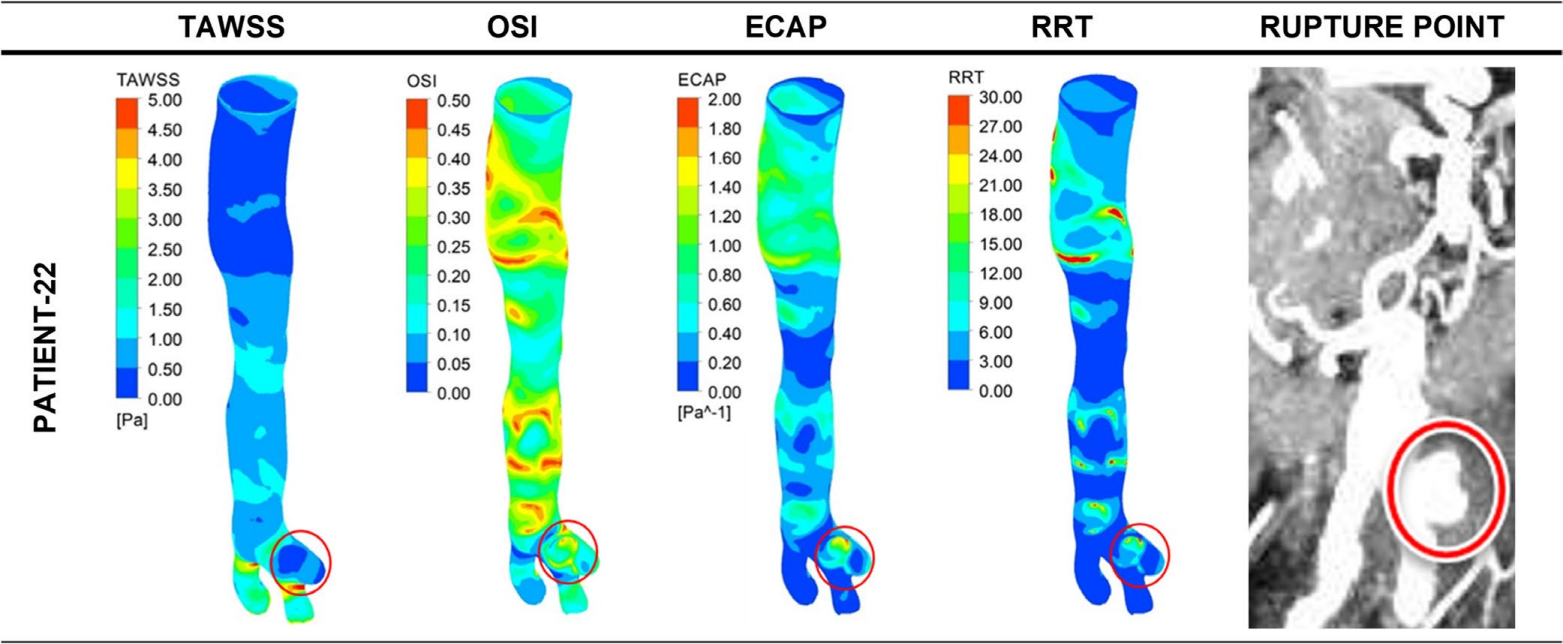
Distribution of WSS parameters of TAWSS, OSI, ECAP, and RRT in contour plots for ruptured saccular high ILT aneurysm cases. The red circles indicate the clinician‐defined rupture site in the CT data and the contour plot area corresponding to the same location.

**FIGURE 10:**
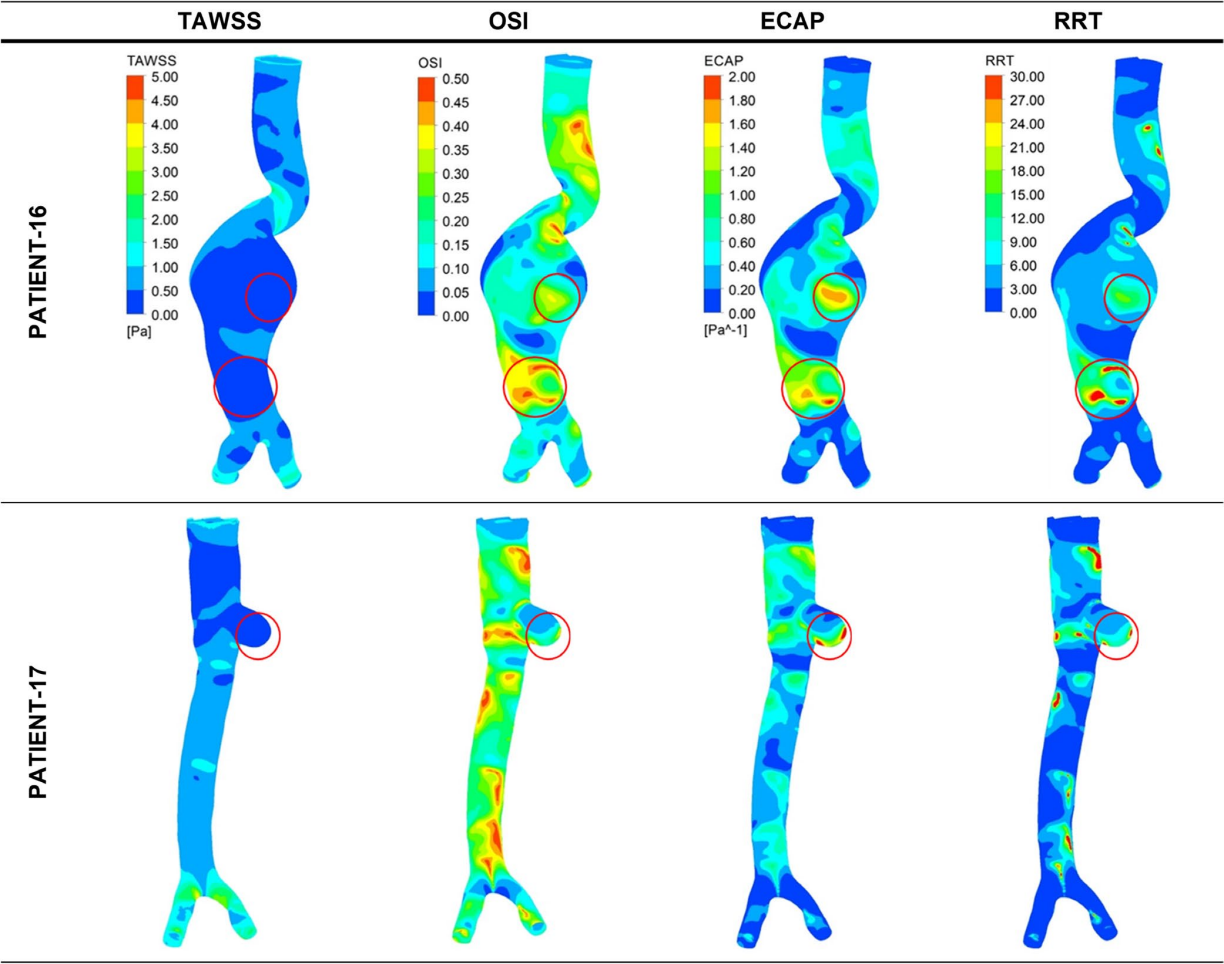
Distribution of WSS parameters of TAWSS, OSI, ECAP, and RRT in contour plots for impending rupture fusiform high ILT aneurysm cases. The red circles indicate the clinician‐defined rupture site in the CT data and the contour plot area corresponding to the same location.

The eight ruptured AAA cases for the high ILT group were divided into two subgroups: fusiform aneurysms and saccular aneurysms. The lower section of Table 3 (patients 13–22) shows the levels of TAWSS, OSI, ECAP, and RRT for all studied cases in the high ILT group. Figure 8 presents the distribution of these parameters in contour plots for 3 representative ruptured fusiform cases (patients 13, 14, and 15), whereas Figure 9 presents these parameters for ruptured saccular cases (Patient 22). The impending ruptured AAA outcomes for the high ILT group are shown in Figure 10 (patients 16 and 17). Additionally, the contour plots for all ruptured fusiform high ILT aneurysm cases (patients 13, 14, 15, 18, 19, 20, and 21) can be seen in Figure S2. The maximum range of TAWSS, OSI, ECAP, and RRT parameters is set to 5 Pa, 0.5, 2 Pa^−1^, and 30, respectively.

**FIGURE 11:**
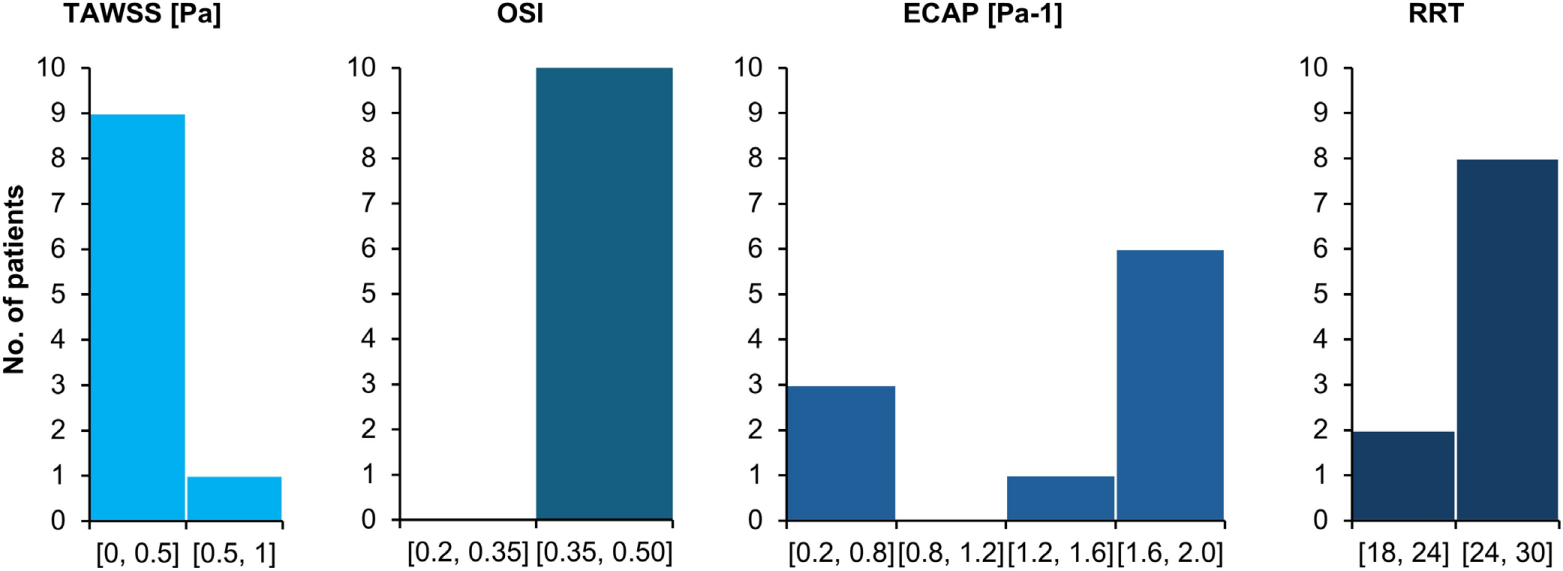
Histograms of TAWSS, OSI, ECAP, and RRT values for patients in the high ILT group.

When the aneurysm rupture sites for all the ruptured cases with high ILT deposition were evaluated, except for patient number 19 from the fusiform aneurysms group, all patients had similar TAWSS values between 0 and 0.5 Pa. All the patients had similar OSI values between 0.35 and 0.50. With the exception of patients 18, 19, 20, and 21, all ECAP values were between 1.60 and 2.00 Pa^−1^; Patient 18 had ECAP values between 1.2 and 1.6 Pa^−1^, while patients 19, 20, and 21 had ECAP values between 0.2 and 0.8 Pa^−1^. Except for patients 20 and 21 from the fusiform group, all patients had similar RRT values between 24 and 30. However, patients 20 and 21 had RRT values between 18 and 24. This distribution of WSS‐related hemodynamic parameters can be more clearly seen in Figure 11 below.

The most notable variation from the expected highest range value trend for the ruptured high ILT cases was seen in the ECAP values. For the fusiform group, patients 13, 14, 15, and 16 had ECAP values in the highest range specified between 1.60 and 2.00 Pa^−1^. However, patients 19, 20, and 21 had ECAP values between 0.20 and 0.80 Pa^−1^, while Patient 18 had ECAP values between 1.2 and 1.6 Pa^−1^. On the other hand, Patient 22 from the saccular group had ECAP values between 1.6 and 2.0 Pa^−1^.

Even though the aneurysms of patients 16 and 17 shown in Figure 10 were for impending rupture cases, at the predicted rupture locations Patient 16 had TAWSS values between 0 and 0.50 Pa, OSI values between 0.35 and 0.50, ECAP values between 1.60 and 2.00 Pa^−1^, and RRT values between 24 and 30, which were comparable to other ruptured fusiform aneurysms with high ILT. Similarly, at the predicted rupture locations, Patient 17 had TAWSS values between 0 and 0.50 Pa, OSI values between 0.35 and 0.50, ECAP values between 1.60 and 2.00 Pa^−1^, and RRT values between 24 and 30, which were comparable to ruptured saccular aneurysms with high ILT.

## 4 Discussion

The rupture of the aortic aneurysm sac is a complex process involving various hemodynamic, biophysical, and biomechanical factors [35, 78]. In this study, disturbed hemodynamics around confirmed rupture sites for AAA cases that were categorized into low and high ILT deposition groups were compared using WSS‐related hemodynamic parameters. Although rupture is the mechanical failure of the arterial structure and is dependent on the mechanical wall stress, the hemodynamic parameters also play an important role in the rupture mechanism [76, 79, 80, 81]. This is due to the close relationship between the flow‐induced shear forces and the arterial remodeling process. The endothelial cells on the arterial wall are influenced by shear forces on the wall and regulate the remodeling of the artery [82]. The abnormal levels of shear stresses on the wall disrupt the proper arterial remodeling process and lead to degeneration in the wall strength [3]. As a result, the weakened artery wall cannot withstand the flow‐induced hemodynamic forces, and AAA rupture occurs.

The WSS‐related parameters of TAWSS, OSI, ECAP, and RRT are critical hemodynamic markers that provide important information on the directionality and magnitude of the shear forces [28]. By determining the contour plots of these parameters on the patient‐specific AAA geometries, a detailed computational investigation was performed to reveal the spatial and temporal distributions of TAWSS, OSI, ECAP, and RRT on the aneurysm sac. By analyzing the aforementioned WSS‐related parameters on the patient‐specific AAA geometries, the actual rupture sites of cases are elucidated in detail and predicted by using the results of CFD simulations. Information about the comorbidities present in the patients was also included, and it was found that, with the exception of a single patient, all the AAA patients had at least two additional coexisting medical conditions. In particular, hypertension was found to be the most common comorbidity present in the considered patients. Since hypertension implies an increased pressure of blood in the body, it is expected that chronic hypertension would contribute significantly to the formation of an AAA, since the sustained rise in pressure would cause the aorta to stretch and weaken over time [83].

According to the retrospective patient‐specific CFD analysis of the already known rupture sites, the TAWSS values at the rupture points are found to be between 0 and 0.50 Pa in almost all patients. There are only two exceptional cases with TAWSS values in the range of 0.50–1 Pa, which is still close to the range of 0–0.50 Pa. The TAWSS values obtained in the current CFD investigations agree with the previously reported TAWSS levels in the literature [3, 30]. The findings suggest that the regions with low TAWSS (particularly between 0 and 0.50 Pa) are potentially high rupture risk sites on the aneurysm sac. This fact is also confirmed by several studies in the literature [31, 34, 40, 45], where the regions with reduced flow velocity and low TAWSS are more prone to rupture.

The levels of OSI at the rupture points are found to be between 0.35 and 0.50 for almost all AAAs. Two exceptional AAAs ruptured with an OSI in the range of 0.20 and 0.35. Theoretically, OSI can have a minimum value of 0 and a maximum value of 0.50, depending on the directionality of the apparent shear forces on the wall. For all the investigated AAAs, the OSI value is high at the rupture points, indicating high variability in the direction of flow‐generated shear forces. This demonstrates that the regions with high OSI (particularly between 0.35 and 0.50 Pa) are the potential regions for eventual AAA rupture. This finding is also supported by the previously performed numerical analyses in the literature, which report an increased rupture risk in the regions with low WSS and high OSI [34, 40, 84].

However, if only regions where the TAWSS values are in the lowest specified range of between 0 and 0.5 Pa, and the OSI values are in the higher specified range of between 0.35 and 0.5 are considered, there may be cases where these criteria are met but the location that satisfies these conditions is not actually a rupture site. This can easily be confirmed by checking the upper section of the contour plots for the healthy patient in Figure 4 of this study. Here, an oval‐shaped region can be observed where these criteria are met, but the region is quite obviously not a rupture site, showing that there may be situations where considering only these two parameters is not adequate. However, this situation changes when all four parameters, TAWSS, OSI, ECAP, and RRT, are taken into consideration.

Although the distribution of ECAP values when looking at all 22 AAA patients is not as clear‐cut as for the TAWSS and OSI values, it was seen that 63.6% of the patients had ECAP values in the highest specified interval between 1.6 and 2.0 Pa^−1^. Likewise, for the RRT values, when all the AAA patients included in this study were considered, about 77.3% of the patients had RRT values in the higher specified range between 24 and 30. Therefore, if the trends in all four of the WSS‐related hemodynamic parameters included in this study are accounted for, the criteria for locating potential rupture sites in an AAA patient would be to look for locations where the TAWSS values are in the lowest specified range of between 0 and 0.5 Pa, the OSI values are in the higher specified range of between 0.35 and 0.5, the ECAP values are in the highest specified interval between 1.6 and 2.0 Pa^−1^, and the RRT values are in the higher specified range between 24 and 30. When these criteria are considered, no corresponding regions were found when observing the contour plots for the healthy patient in Figure 4. This finding is also supported by previously performed numerical analyses in the literature, which report an increased rupture risk in regions with low TAWSS, high OSI, high ECAP, and high RRT [85]. Further confirmation of this can be obtained when looking at non‐rupture locations for individual patients included in this study. For example, when observing the AAA contour plots for patients 13, 14, and 22, there are multiple regions that are within the above specified thresholds that are indicative of rupture sites, for up to three of the four considered parameters. However, when all four parameters, TAWSS, OSI, ECAP, and RRT, are concurrently analyzed, these sites can then be ruled out as possible rupture locations. Sites within the specific ranges: TAWSS 0–0.5 Pa, OSI 0.35–0.5, ECAP 1.6–2.0 Pa^−1^, RRT 24–30, essentially represent a localized “hemodynamic perfect storm” where a combination of sufficiently static, and extremely oscillatory flow enhances the degradation of the arterial wall, while at the same time allowing for prolonged exposure to harmful chemicals and inflammatory agents, which weaken the artery wall.

When the results are analyzed while looking at the distribution of the four WSS‐related hemodynamic parameters for the patients in the no/low ILT group and the patients in the high ILT group, respectively, the general trend of the results in both groups agrees with the overall trend observed for all the patients. Further confirmation of this can be attained by observing the histograms in Figures 7 and 11, for which the shape of the distribution for the four parameters is strikingly similar. This indicates that, according to the results of this study, the degree of ILT deposition did not significantly affect the distribution of the values for TAWSS, OSI, ECA, and RRT. Similar cases have been found in previous studies, where there did not seem to be a correlation between the values of hemodynamic parameters and the degree of ILT deposition [41, 46]. On the other hand, when the comorbidities present in the patients were considered, it was observed that there was generally a higher percentage of patients in the high ILT group affected by coexisting medical conditions. Therefore, the increased presence of the reported comorbidities in the patients seems to lead to a higher susceptibility to ILT formation.

According to the results of CFD simulations, the general trend observed for all the patients in TAWSS, OSI, ECAP, and RRT levels at the rupture points is also found to be nearly unchanged when looking at only fusiform cases. This is not surprising, as of the 22 AAA patients considered in this study, 18 of them exhibited fusiform AAAs. On the other hand, for the four saccular AAA cases examined, there was a break from the general trend. For all four cases, the TAWSS values were still in the lowest specified range of between 0 and 0.5 Pa. However, for the OSI values, there was a patient out of the four whose values were observed to be between the lower specified interval of 0.2 to 0.35. Although this is limited to a single patient, this means that when considering only the saccular group of patients 75% of the patients had OSI values in the higher specified range of 0.35 and 0.5, which is a lower percentage value than for all the cases as a whole, the no/low ILT group, the high ILT group, and the fusiform only group respectively. This seems to suggest that for saccular aneurysms, the variability in the direction of flow‐generated shear forces is not as high as for the other groups. On the other hand, 75% of the patients in the saccular aneurysm group had ECAP values in the highest specified range of 1.6–2.0 Pa^−1^, which was a percentage in reasonable agreement with the values found for the other groups. Additionally, all four of the patients with saccular aneurysms had RRT values between the higher specified interval of 24–30.

## 5 Conclusion

Given the current uncertainty surrounding the prediction of AAA rupture based solely on diameter and growth rate, this study investigates a comprehensive and integrated approach for the prediction of potential rupture locations in the 22 available patient‐specific AAA geometries through the analysis of the values of the four WSS‐derived parameters TAWSS, OSI, ECAP, and RRT obtained from CFD simulations. Giving consideration to the data available in routine clinical practice, the geometries obtained from CT scans were modeled with standard boundary conditions, and a rigid‐wall assumption was adopted. Even with these limitations, the spatial distributions of TAWSS, OSI, ECAP, and RRT were clearly heterogeneous, mainly due to the high complexity in the patient‐specific AAA geometries [28]. These complex geometries result in many local minima and maxima for the hemodynamic parameters on the AAA sac. The following are the key findings obtained in this study:

- The rupture sites in the AAAs of the examined patients were found to correspond most closely with the regions where the values for the WSS‐related hemodynamic parameters were between 0 and 0.5 Pa for TAWSS, between 0.35 and 0.5 for OSI, between 1.6 and 2.0 Pa^−1^ for ECAP, and between 24 and 30 for RRT.
- The simultaneous analysis of all four of these hemodynamic parameters was found to be critical for the accurate assessment of rupture risk, as considering only TAWSS and OSI can lead to flawed predictions.
- The degree of ILT (no/low ILT versus high ILT) did not significantly alter the trends observed in the values of the WSS‐related hemodynamic parameters, although patients within the high ILT group had a higher prevalence of the assessed comorbidities.
- The trend observed for all the patients as a whole in the WSS‐related hemodynamic parameters was maintained when considering only patients with fusiform aneurysms. However, there was some deviation from this trend observed for the saccular AAAs, particularly in OSI and RRT values.

Overall, the study shows that analyzing the TAWSS, OSI, ECAP, and RRT values obtained from CT‐based CFD simulations that employ standardized boundary conditions can play a crucial role in assessing the risk of rupture in AAA patients. In addition, the simplified setup employed substantially reduces the pre‐processing time and computational cost that would be involved when incorporating CFD into clinical practice.

However, the generalizability of the findings is not as well supported for the saccular aneurysm cases due to the relatively small number of cases. Future research work can focus on expanding the sample size to further validate these findings across a larger, more diverse patient cohort, which includes more healthy patients, more patients with cases of saccular aneurysms, and patients with AAAs that are not at a stage where surgical intervention is required. Furthermore, future research work can investigate the robustness of the simplified modeling approach taken in this study against approaches that employ more complexity to offer greater physical fidelity. This may include the inclusion of patient‐specific boundary conditions and the incorporation of fluid–structure interaction (FSI) to model patient‐specific wall mechanics, for example. Nevertheless, it should be noted that in order to obtain accurate boundary conditions and arterial wall properties, additional medical procedures, besides CTA, would need to be conducted. For the comprehensive visualization of flow patterns, a dependable method would be the inclusion of 4D Flow MRI [86]. Whereas, in order to determine patient‐specific material properties for the aorta wall, 4D ultrasound would be an effective technique [87]. On the other hand, it would be expected that with an increase in the number of patients and complexity considered, the computational load required for CFD simulations may become excessive. When future clinical applications are contemplated, this can develop into a significant hurdle, especially when the time‐sensitive nature of decisions to be made is taken into account. Therefore, the integration of deep learning in order to streamline the analysis of WSS‐related parameters of AAAs is also a worthwhile avenue for future research [88].

Moreover, due to the relatively large patient cohort considered in this study, it was decided to focus on the quantitative assessment of WSS‐related hemodynamic parameters in AAAs. Future studies can also qualitatively assess the important features related to disturbed hemodynamics, such as flow stasis, recirculation zones, and vortical structures.

## Author Contributions

All authors contributed to the study conception and design. O.M. was responsible for constructing CFD geometries from patient CT data, conducting simulations, and compiling the first draft. H.E.S. assisted with the figure preparation, compilation, and refining of the manuscript. A.E.‐M. contributed clinical expertise, particularly in the identification of rupture sites from patient CT data and the categorization of patient AAAs. M.M.Y. and M.E.H.C. facilitated the design and implementation of the computational workflow, providing methodological support. R.A.K. contributed during the development of the final manuscript by conducting the mesh sensitivity analysis, reorganizing the results, generating new data visualizations, and revising the discussion and conclusion sections to enhance scientific interpretation and readability. H.A.‐T. recruited the patients, obtained medical images, provided clinical guidance, and reviewed relevant parts of the manuscript. H.C.Y. initiated the investigation and assembled the project team, secured ethical approvals and funding, trained and supervised the Research Assistants (RAs) for the execution of the work, created the outline of the paper, and provided close oversight and guidance to the RAs during the drafting, revision and finalization of the manuscript. Additionally, H.A.‐T. and H.C.Y. offered critical feedback throughout, and ensured the overall integrity of the research. All authors contributed to manuscript editing and approved the final version.

## Funding

This work was funded by the Qatar National Research Fund (QNRF), National Priority Research Program (Grant number NPRP13S‐0108‐200024) to H.C.Y. and O.M., Qatar University International Research Collaboration Co‐Funds (IRCC) Program (IRCC 2020‐002) to H.C.Y., and TÜBİTAK (The Scientific and Technological Research Council of Türkiye) 3501—Career Development Program (Project number: 221M001) to H.E.S., Qatar University Graduate Assistantship Award (ID 378) to R.A.K. The authors would like to thank Qatar University for funding this work. Open‐access funding is provided by the Qatar National Library.

## Ethics Statement

IRB approval was received from Hamad Medical Center, Doha, Qatar, Protocol Number: MRC‐02‐20‐134.

## Conflicts of Interest

The authors declare no conflicts of interest.

## Supporting information


**Figure S1:** Distribution of WSS parameters of TAWSS, OSI, ECAP, and RRT in contour plots for healthy abdominal aorta and *all* ruptured fusiform low ILT aneurysm cases (patients 1, 3, 5, 6, 7, 8, 9, 10, and 11). The red circles indicate the clinician‐defined rupture site in the CT data and the contour plot area corresponding to the same location.
**Figure S2:** Distribution of WSS parameters of TAWSS, OSI, ECAP, and RRT in contour plots for *all* ruptured fusiform high ILT aneurysm cases (patients 13, 14, 15, 18, 19, 20, and 21). The red circles indicate the clinician‐defined rupture site in the CT data and the contour plot area corresponding to the same location.

## Data Availability

The data that supports the findings of this study are available in the Supporting Information of this article.
