# Supplementary material for: Integrated Assessment of Wall Shear Stress‐Related Hemodynamic Parameters in Abdominal Aortic Aneurysms: A Retrospective Cross‐Sectional Study on Ruptured Cases
Source: Int J Numer Method Biomed Eng. 2026 Mar 20;42(3):e70153. doi: 10.1002/cnm.70153 (PMC13003724; doi:10.1002/cnm.70153)
Supplement: Supplementary file 1 — Figure S1: Distribution of WSS parameters of TAWSS, OSI, ECAP, and RRT in contour plots for healthy abdominal aorta and all ruptured fusiform low ILT aneurysm cases (patients 1, 3, 5, 6, 7, 8, 9, 10, and 11). The red circles indicate the clinician‐defined rupture site in the CT data and the contour plot area corresponding to the same location. Figure S2: Distribution of WSS parameters of TAWSS, OSI, ECAP, and RRT in contour plots for all ruptured fusiform high ILT aneurysm cases (patients 13, 14, 15, 18, 19, 20, and 21). The red circles indicate the clinician‐defined rupture site in the CT data and the contour plot area corresponding to the same location. [file CNM-42-e70153-s001.docx]

**Supporting Information - Integrated Assessment of Wall Shear Stress-Related Hemodynamic Parameters in Abdominal Aortic Aneurysms: A Retrospective Cross-Sectional Study** **on Ruptured Cases**

Onur Mutlu^1#^, Rahib Ahmed Khan^1,2#^, Huseyin Enes Salman^3^, Ayman El-Menyar^4,5^, Mehmet Metin Yavuz^6^, Muhammad E.H. Chowdhury^7^, Hassan Al-Thani^4*^, Huseyin Cagatay Yalcin^1,2, 8^*

^1^Biomedical Research Center, Qatar University P.O. Box 2713, Doha, Qatar

^2^Department of Mechanical and Industrial Engineering, College of Engineering, Qatar University P.O. Box 2713, Doha, Qatar

^3^Department of Mechanical Engineering, TOBB University of Economics and Technology, Ankara 06560, Turkey

^4^Department of Surgery, Trauma and Vascular Surgery, Hamad General Hospital, Hamad Medical Corporation P.O. Box 3050, Doha, Qatar

^5^Clinical Medicine, Weill Cornell Medicine P.O. Box 24144, Doha, Qatar

^6^Department of Mechanical Engineering, Middle East Technical University, Ankara 06800, Turkey

^7^Department of Electrical Engineering, College of Engineering, Qatar University P.O. Box 2713, Doha, Qatar

^8^Department of Biomedical Science, College of Health Sciences, Qatar University P.O. Box 2713, Doha, Qatar

^#^Equal contributor

*Corresponding authors: Halthani@hamad.qa, hyalcin@qu.edu.qa

E-mail list of authors: onur.mutlu@qu.edu.qa, rkhan@qu.edu.qa, hsalman@etu.edu.tr, aelmenyar@hamad.qa, ymetin@metu.edu.tr, mchowdhury@qu.edu.qa, Halthani@hamad.qa, hyalcin@qu.edu.qa

The following equations were input as expressions in Ansys CFX to accurately replicate the physiological velocity and pressure profiles used in this study, the graphs of which can be seen in Figure 1d:

Velocity = 0.0936904*sin(3.72789*x+0.451644)[m/s] + 0.0545888*sin(17.4208*x+-3.05102)[m/s] + 0.0788792*sin(27.2541*x+0.0368363)[m/s] + 0.104707*sin(10.4327*x+-0.994182)[m/s] + 0.0764448*sin(28.6534*x+2.35068)[m/s] + 0.00835602*sin(38.4819*x+-1.21175)[m/s] + 0.00396129*sin(46.6582*x+-3.66449)[m/s]; where x was varied from 0 to 1 in each cardiac cycle (1)

Pressure = 21623.2*sin(2.92021*p+0.0331885)[Pa] + 7703.93*sin(5.5202*p+1.64304)[Pa] + 846.709*sin(12.1704*p+2.1353)[Pa] + 1124.27*sin(18.7255*p+1.66699)[Pa] + 930.864*sin(24.5466*p+1.24334)[Pa] + 146125*sin(30.841*p+4.05785)[Pa] + 146528*sin(30.8294*p+0.92368)[Pa]; where p was varied from 0 to 1 in each cardiac cycle (2)

These expressions were then utilized when setting up the boundary conditions for the simulations. More detailed information can be found in Section 2.4 of the paper.

|  | **TAWSS** | **OSI** | **ECAP** | **RRT** | **RUPTURE POINT** |
| --- | --- | --- | --- | --- | --- |
| **HEALTHY ABDOMINAL AORTA** | 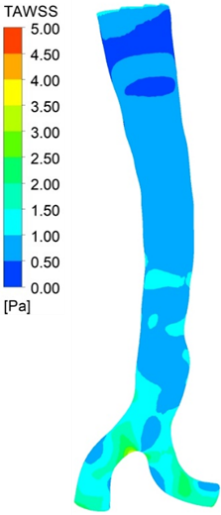 | 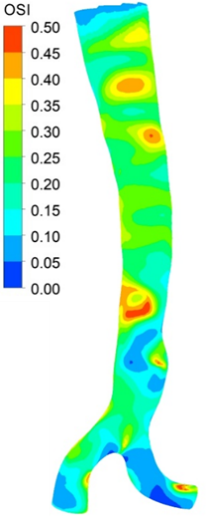 | 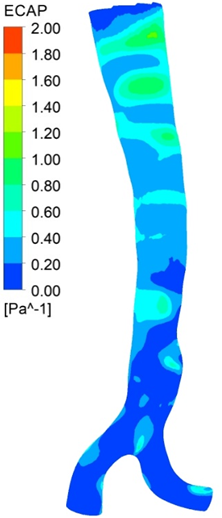 | 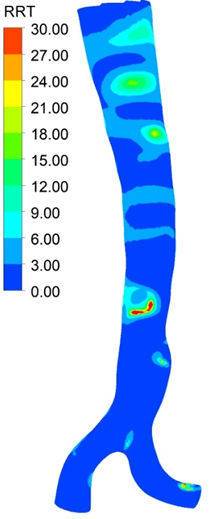 | N/A |
| **PATIENT-1** | 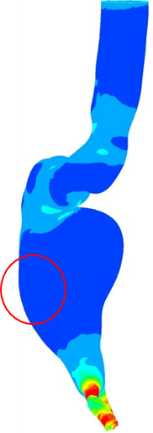 | 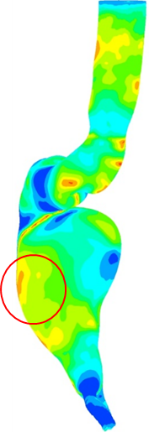 | 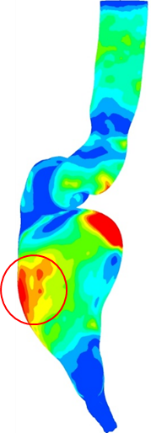 | 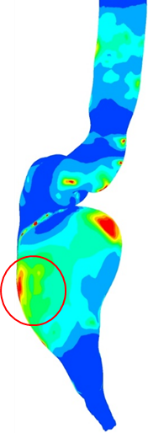 | 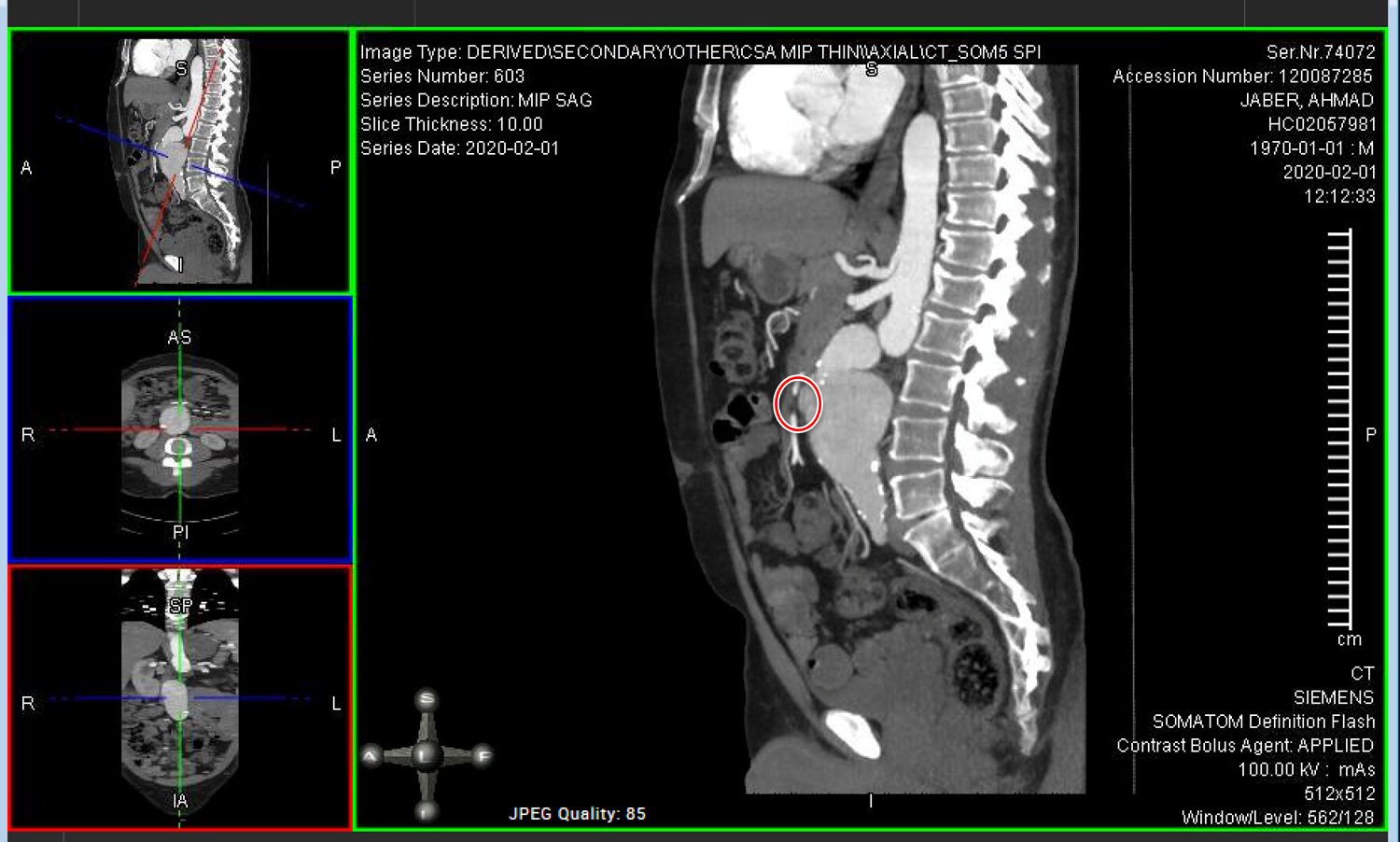 |
| **PATIENT-3** | 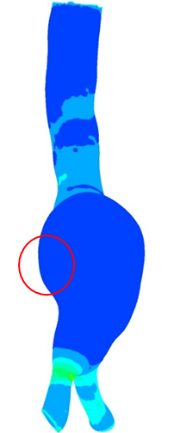 | 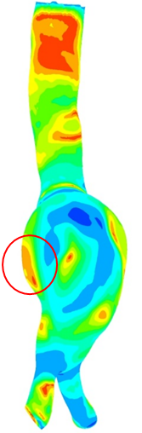 | 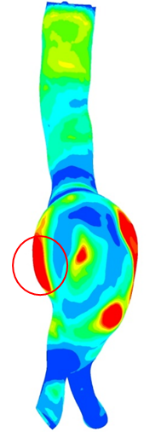 | 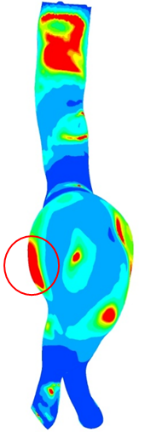 | 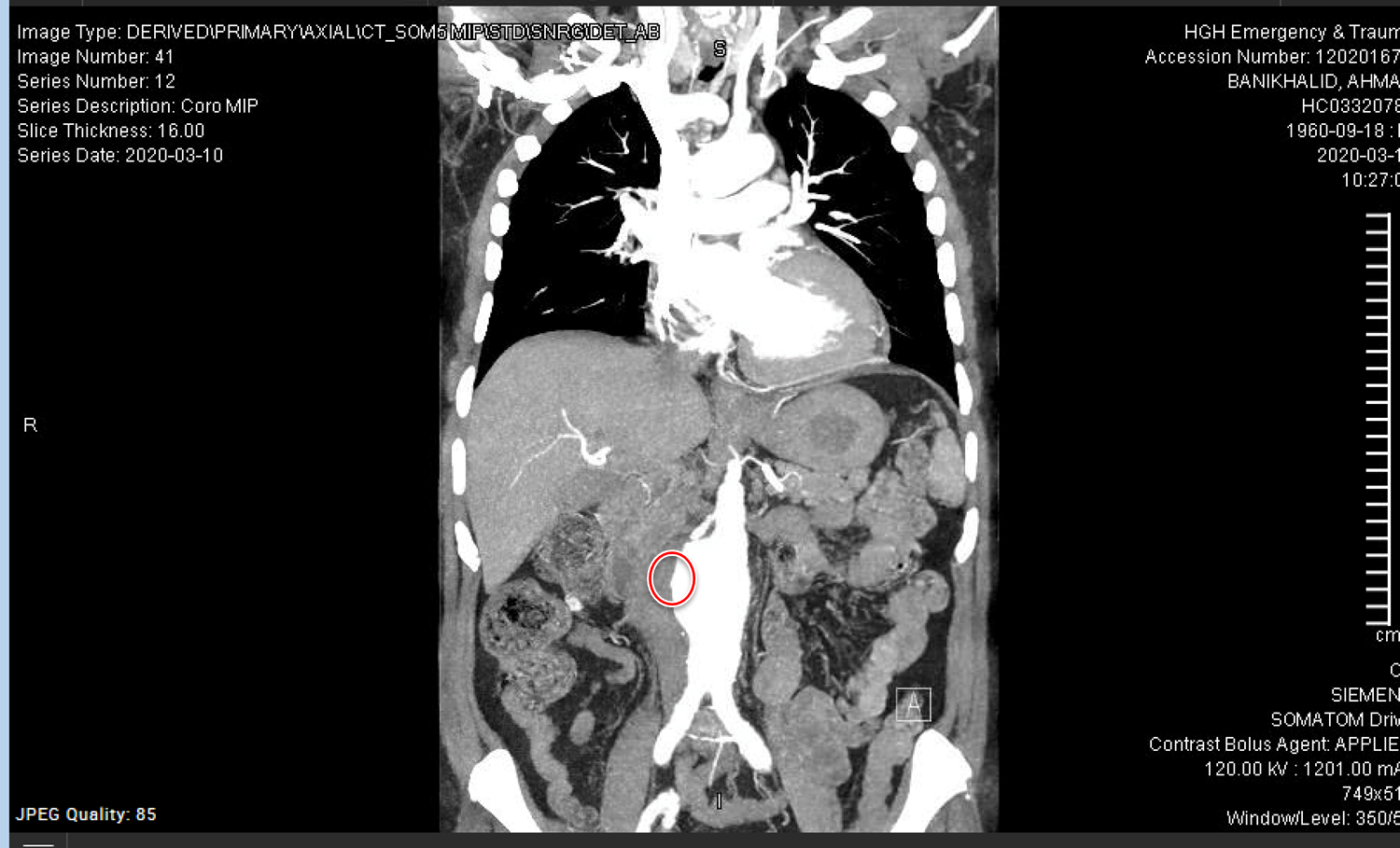 |
| **PATIENT-5** | 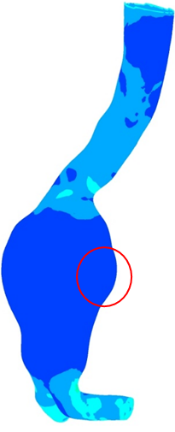 | 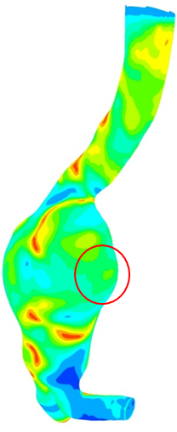 | 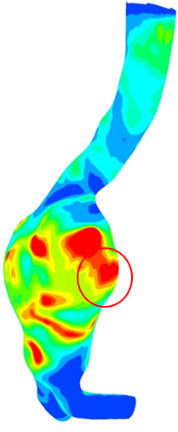 | 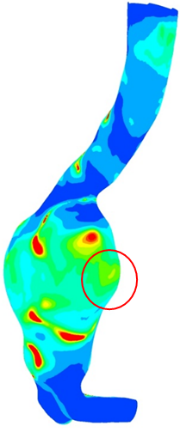 | 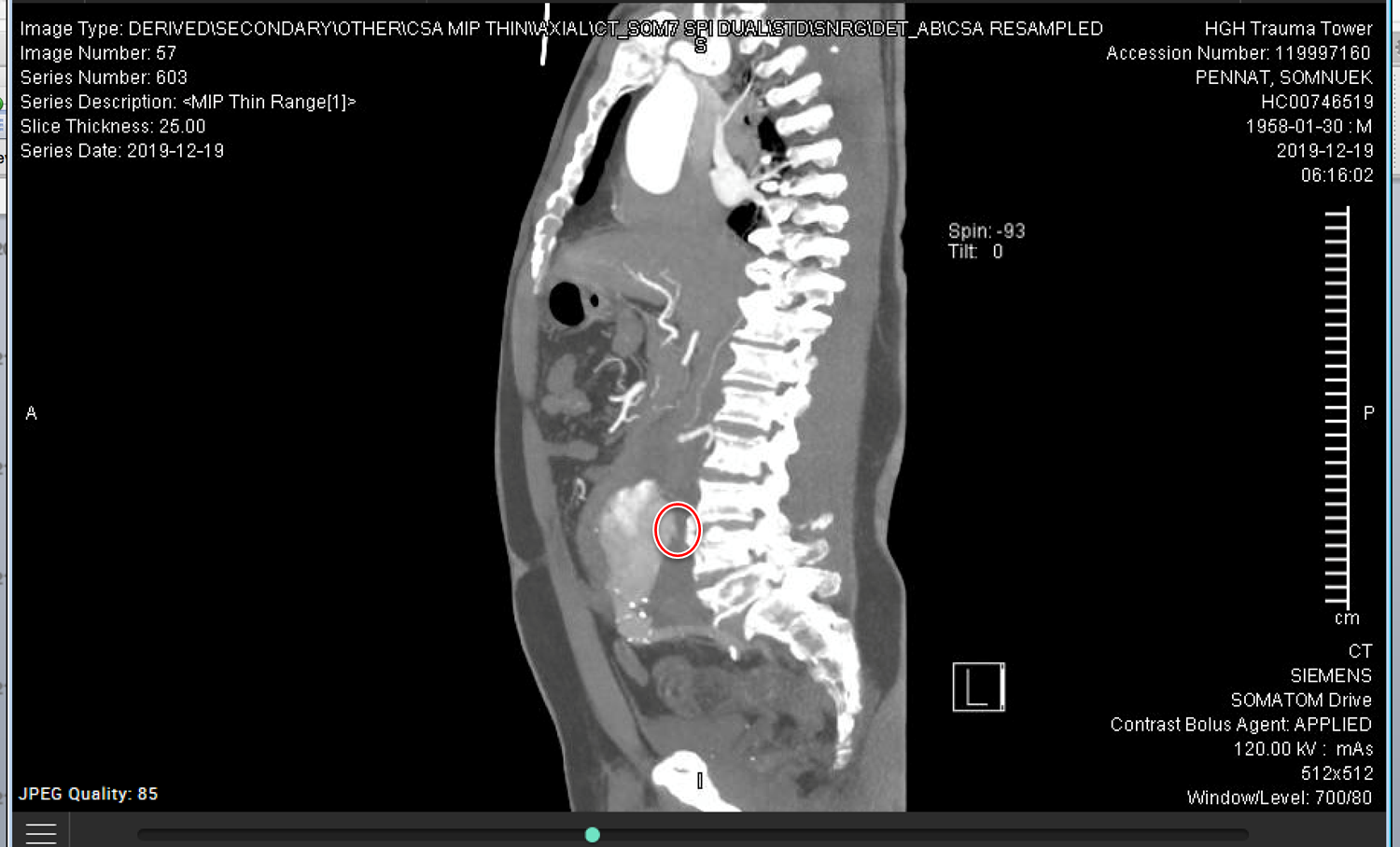 |
| **PATIENT-6** | 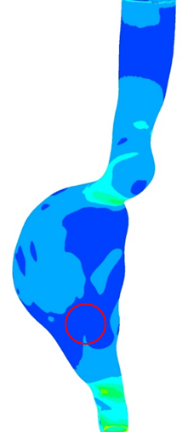 | 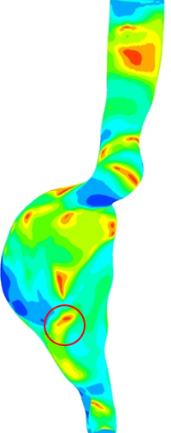 | 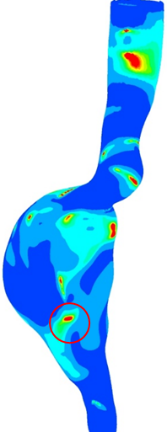 | 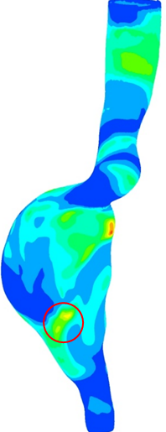 | 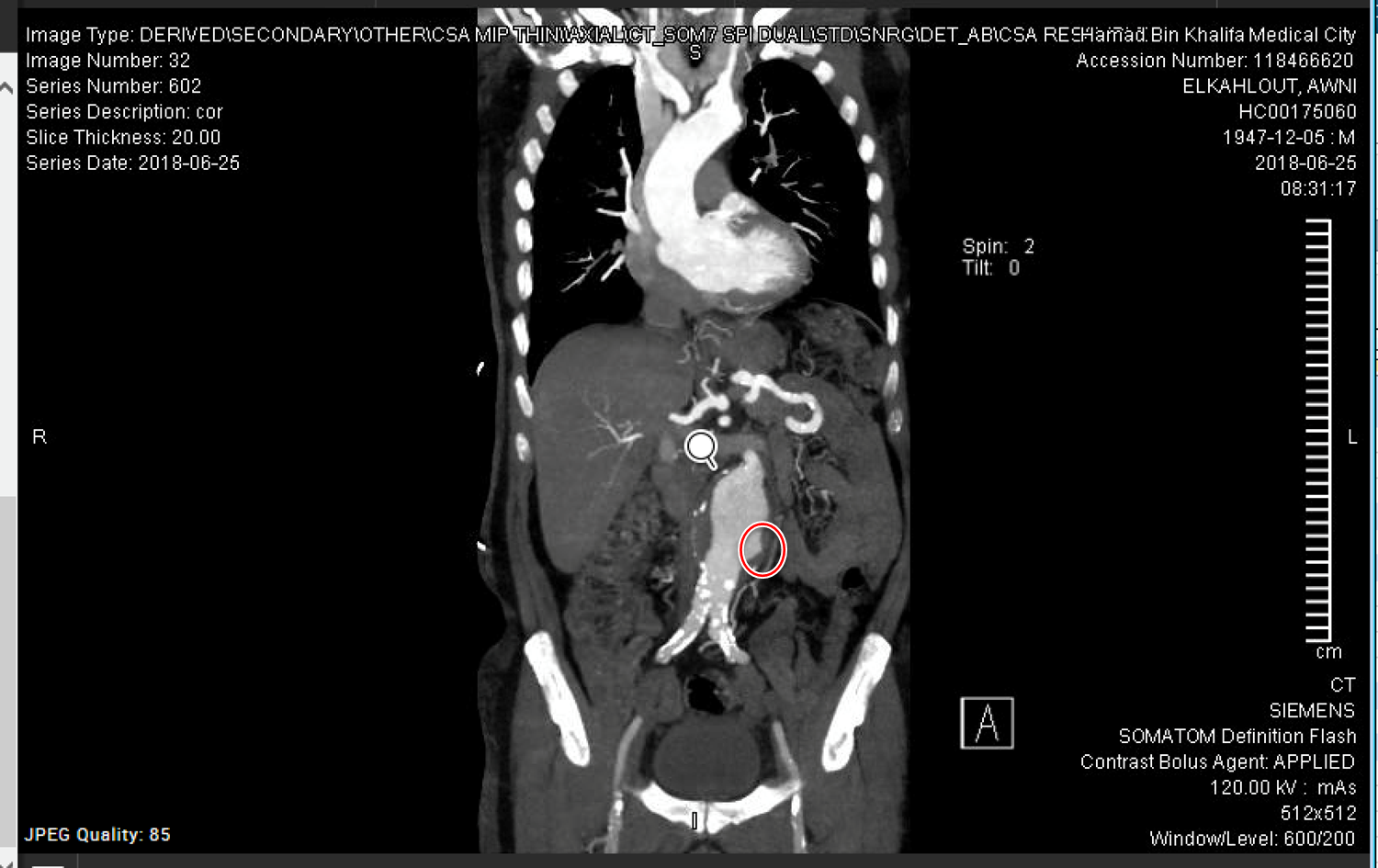 |
| **PATIENT-7** | 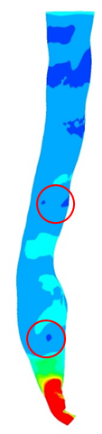 | 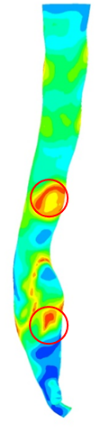 | 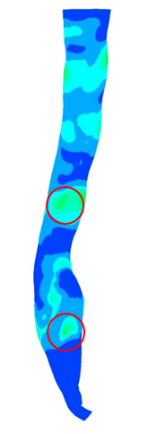 | 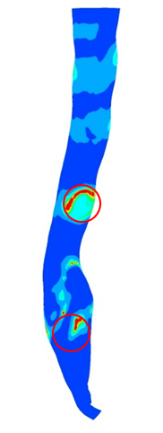 | 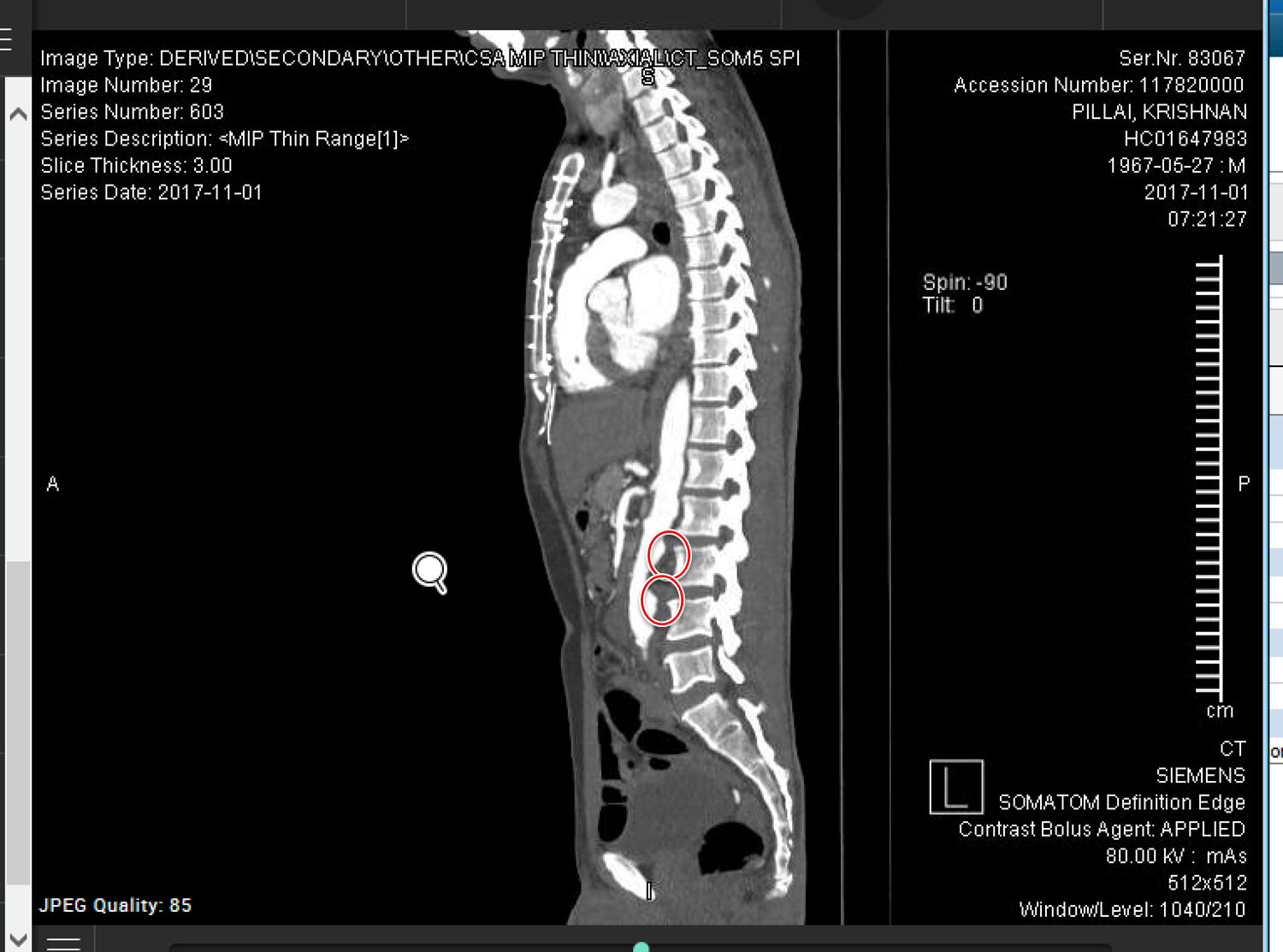 |
| **PATIENT-8** | 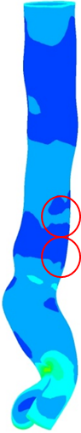 | 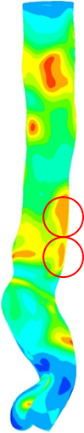 | 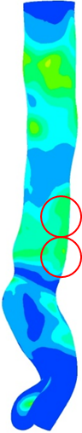 | 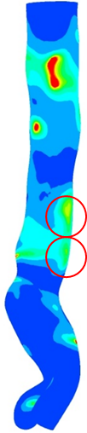 | 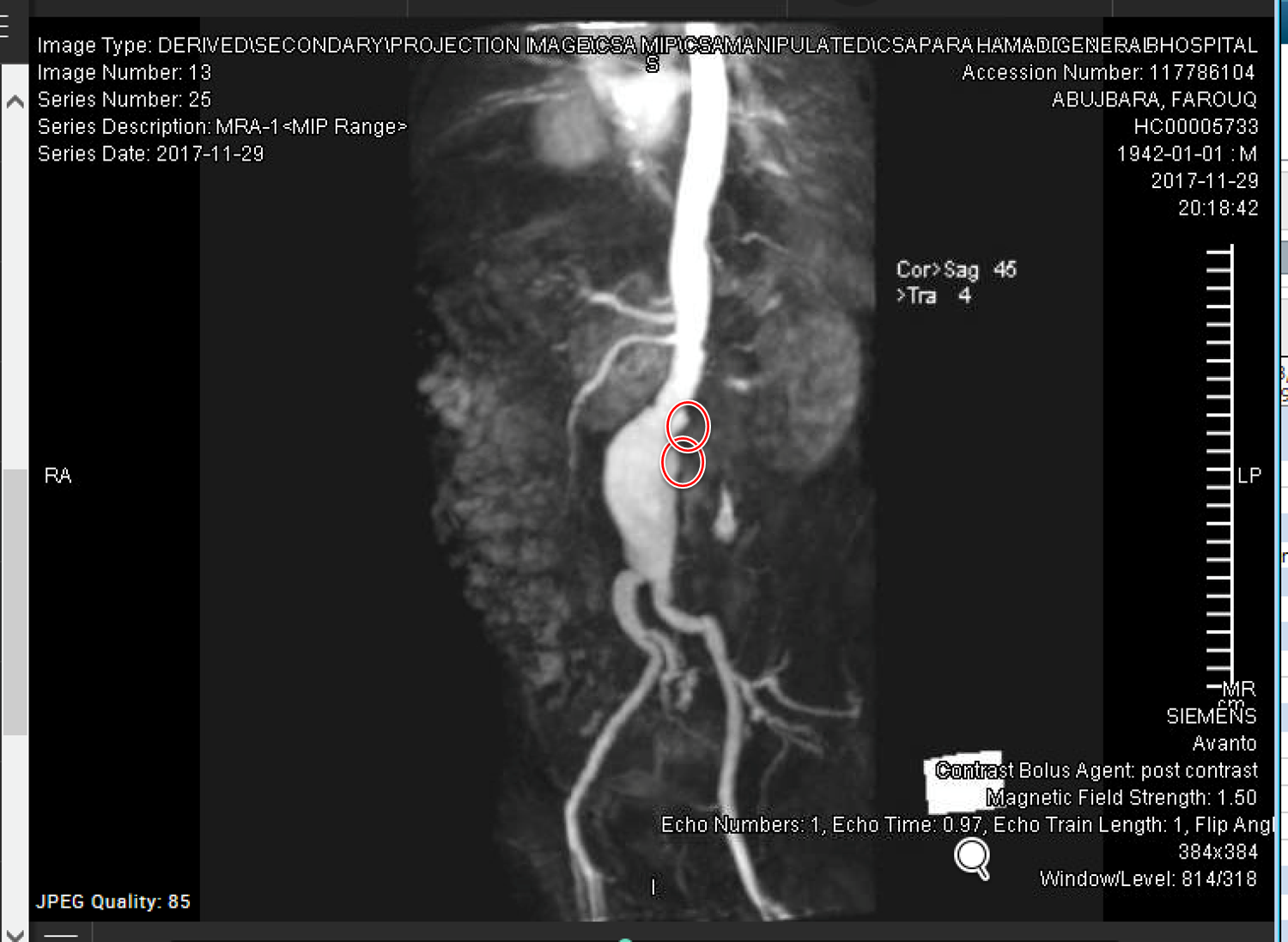 |
| **PATIENT-9** | 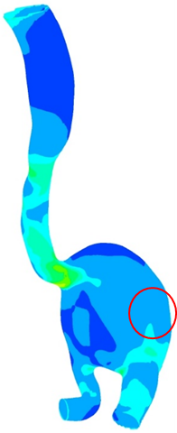 | 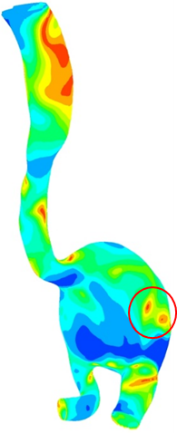 | 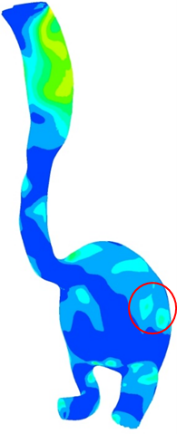 | 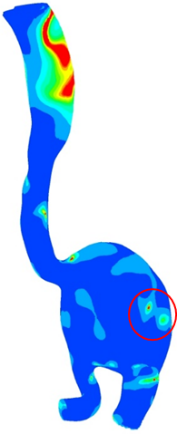 | 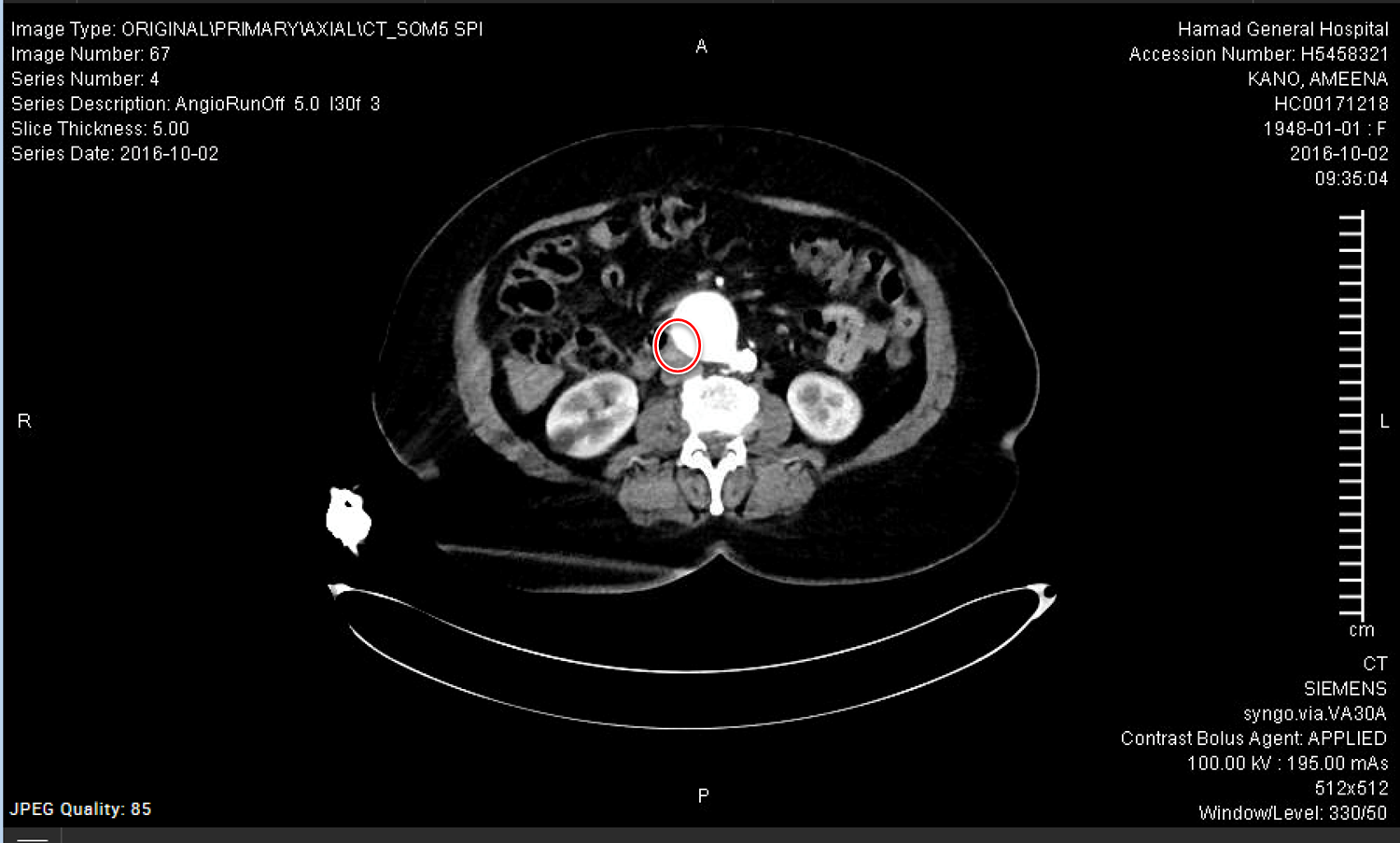 |
| **PATIENT-10** | 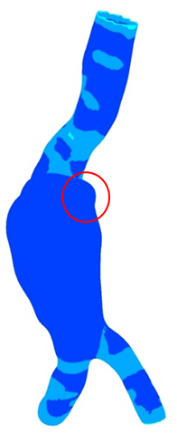 | 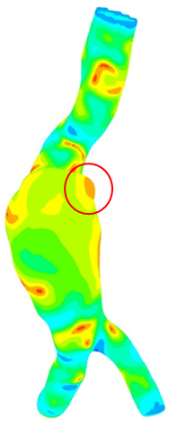 | 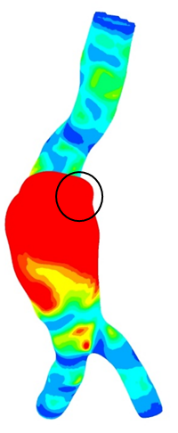 | 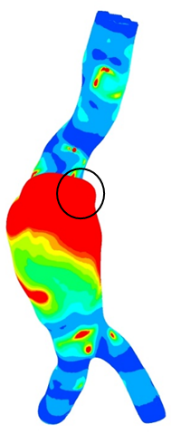 | 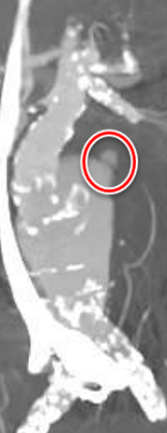 |
| **PATIENT-11** | 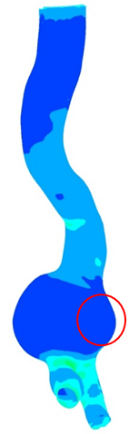 | 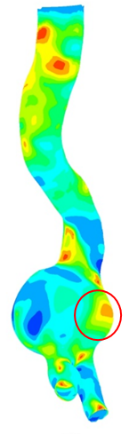 | 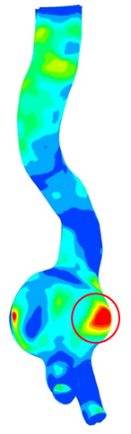 | 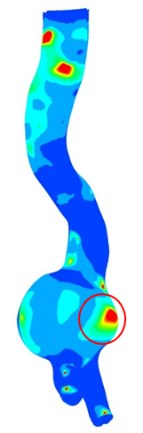 | 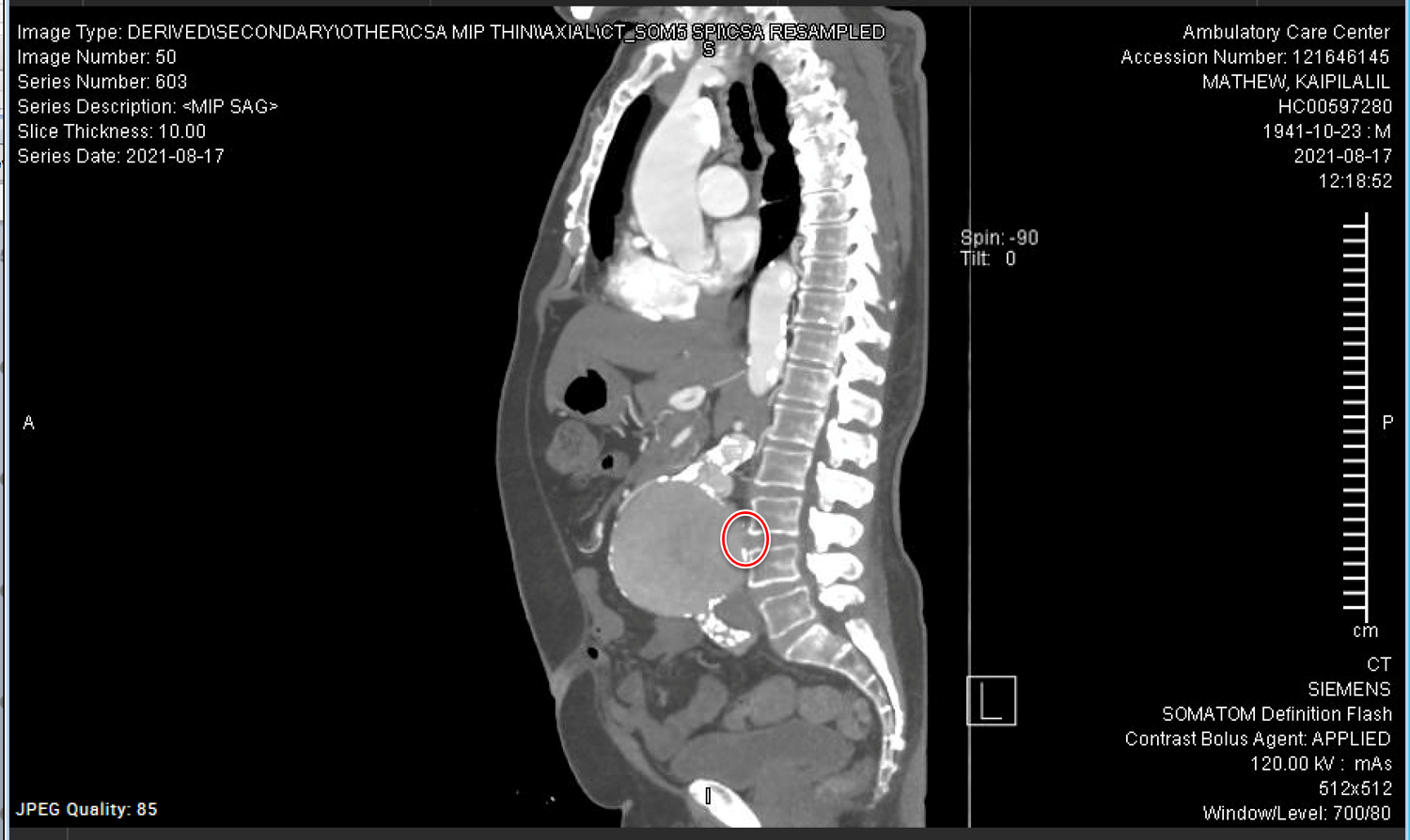 |

**FIGURE S1** Distribution of WSS parameters of TAWSS, OSI, ECAP, and RRT in contour plots for healthy abdominal aorta and **all** ruptured fusiform low ILT aneurysm cases (patients 1, 3, 5, 6, 7, 8, 9, 10, and 11). The red circles indicate the clinician-defined rupture site in the CT data and the contour plot area corresponding to the same location.

|  | **TAWSS** | **OSI** | **ECAP** | **RRT** | **RUPTURE POINT** |
| --- | --- | --- | --- | --- | --- |
| **PATIENT-13** | 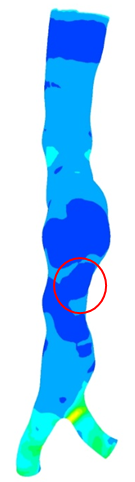 | 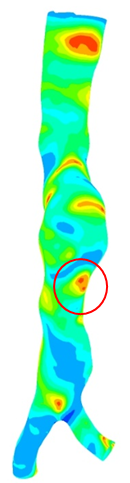 | 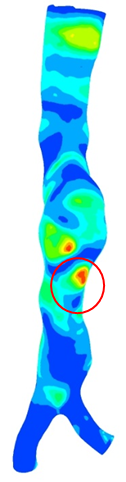 | 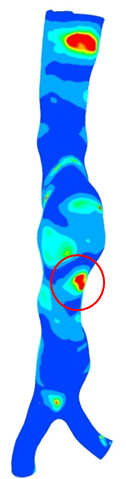 | 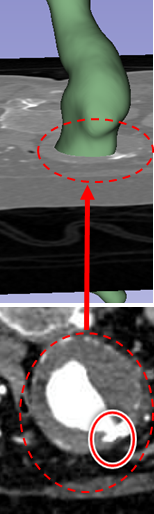 |
| **PATIENT-14** | 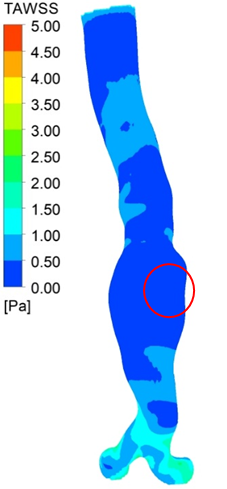 | 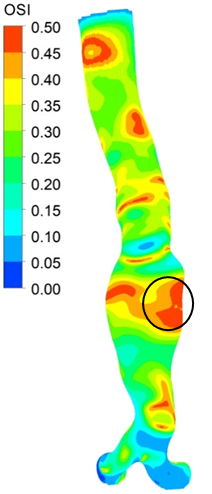 | 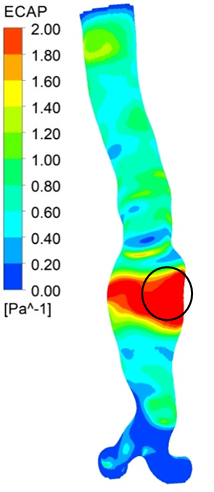 | 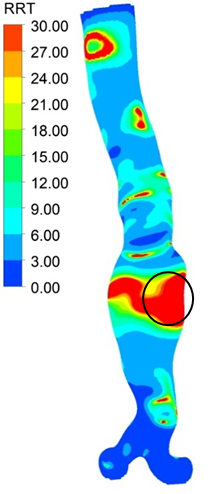 | 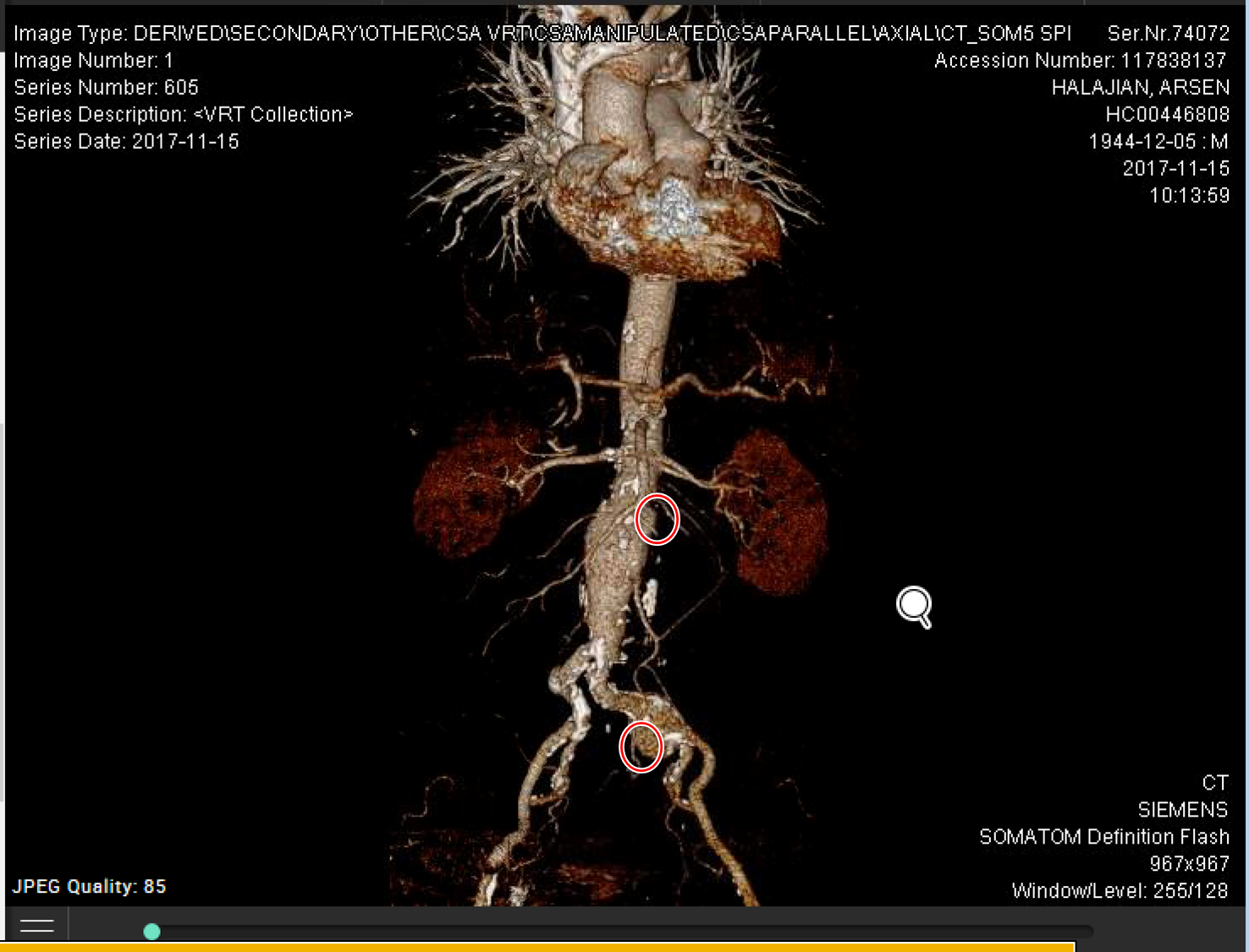 |
| **PATIENT-15** | 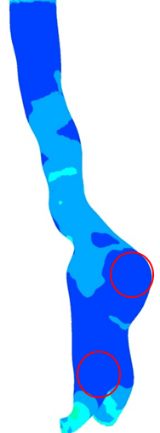 | 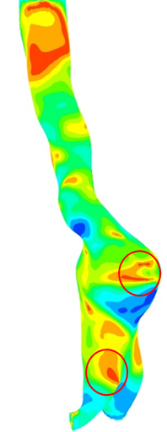 | 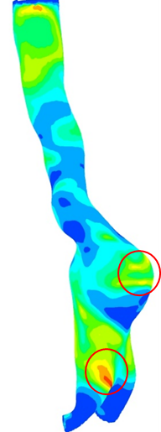 | 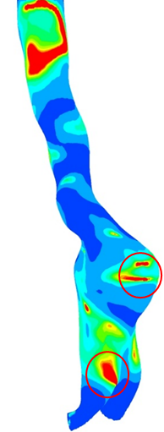 | 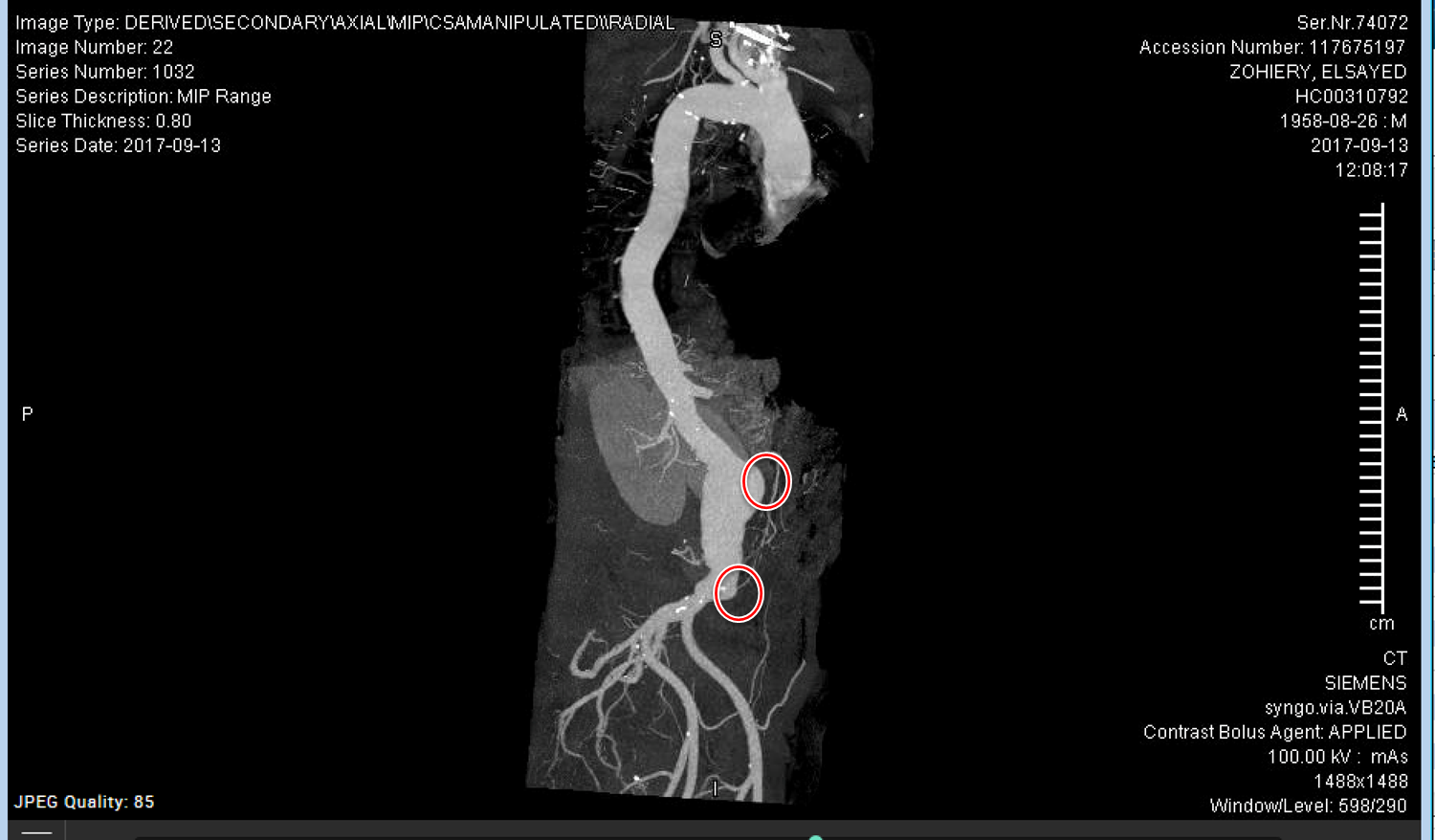 |
| **PATIENT-18** | 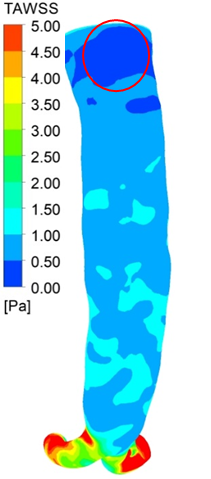 | 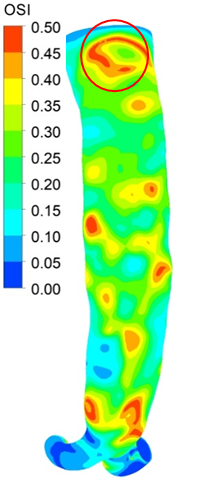 | 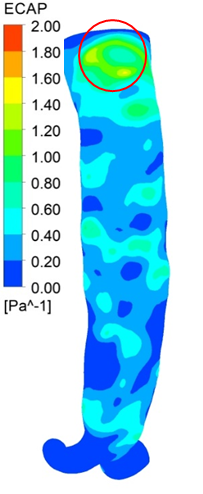 | 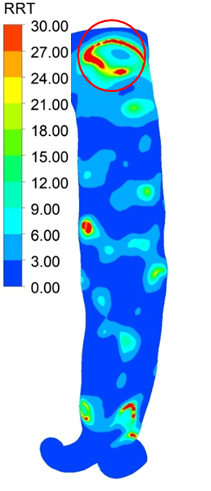 | 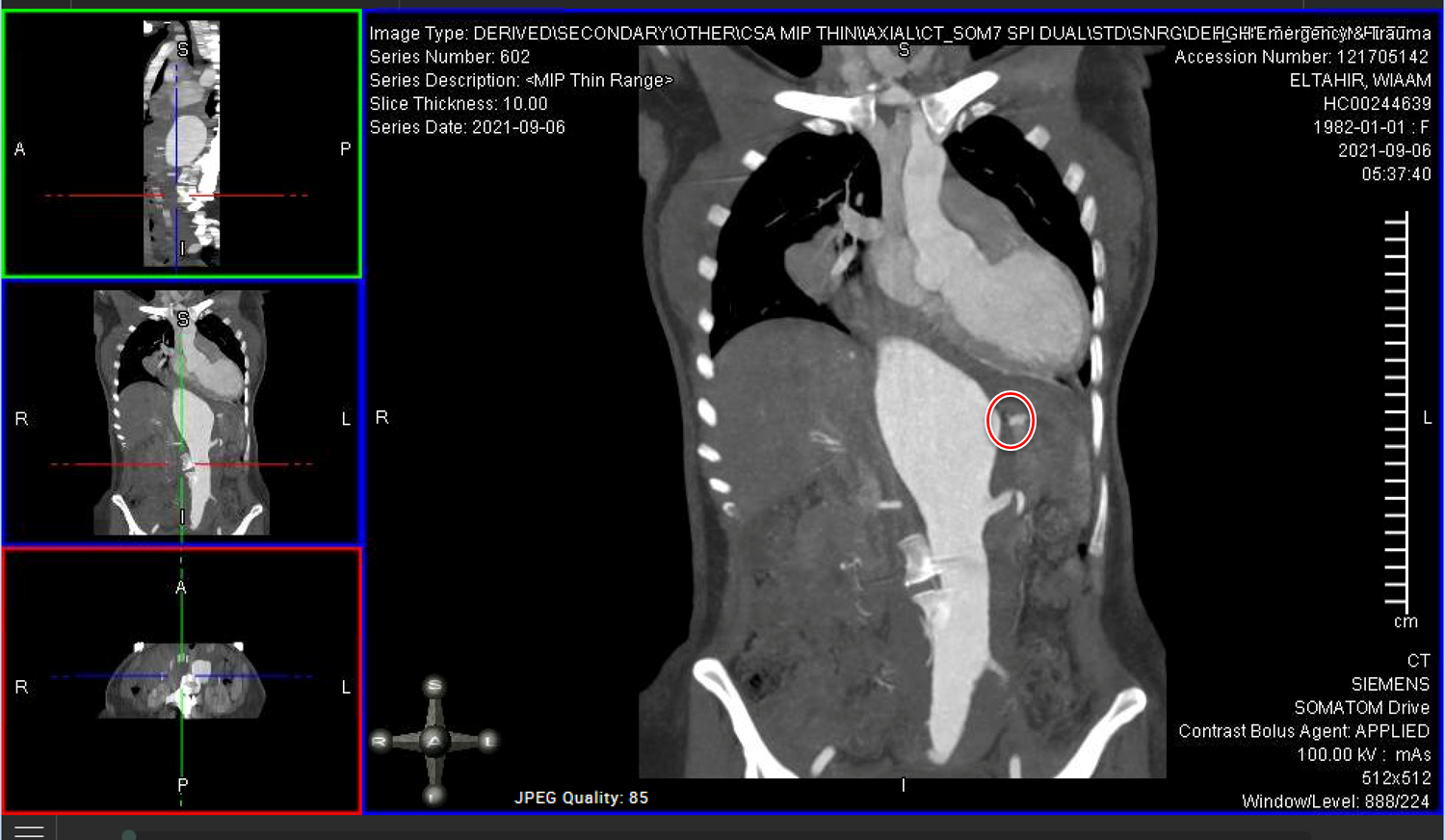 |
| **PATIENT-19** | 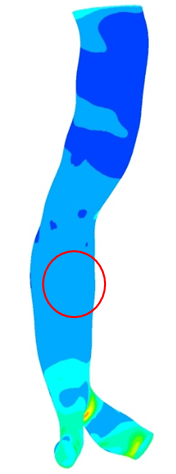 | 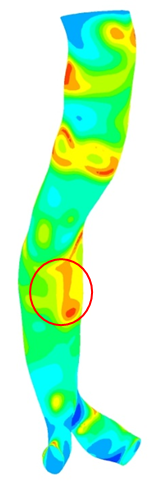 | 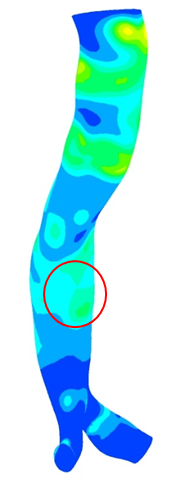 | 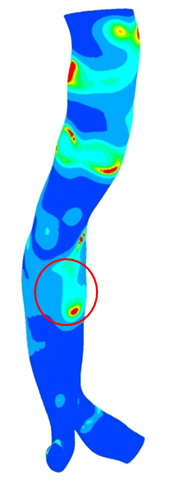 | 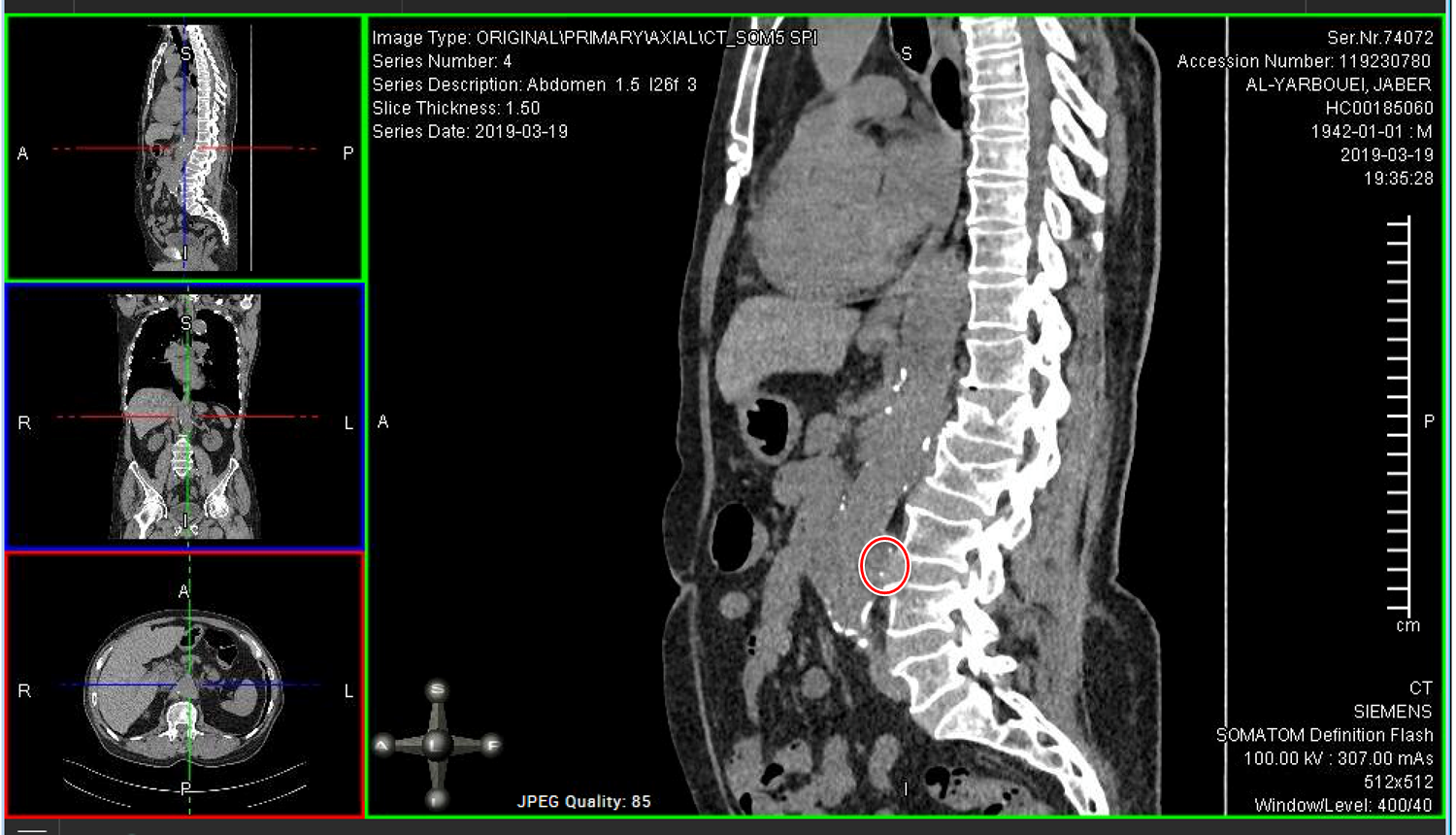 |
| **PATIENT-20** | 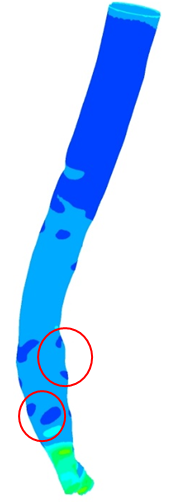 | 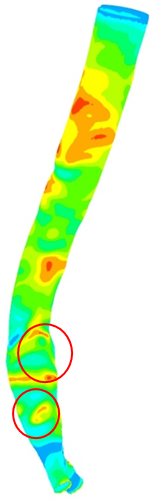 | 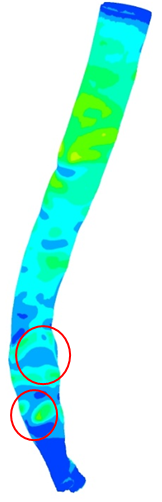 | 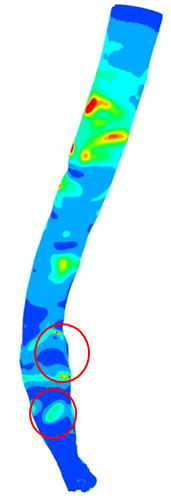 | 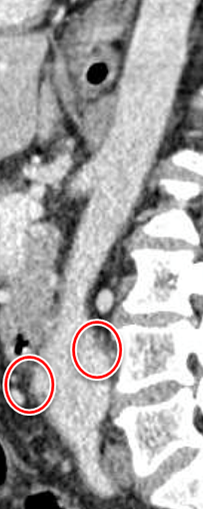 |
| **PATIENT-21** | 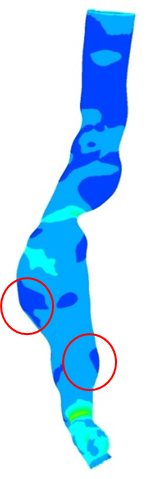 | 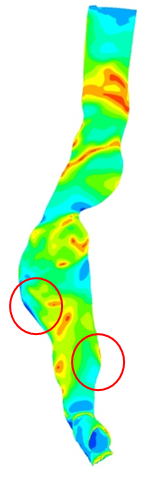 | 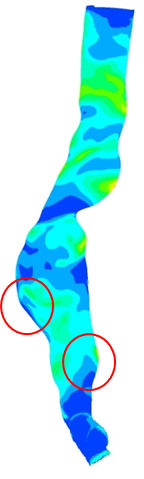 | 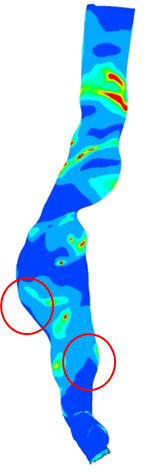 | 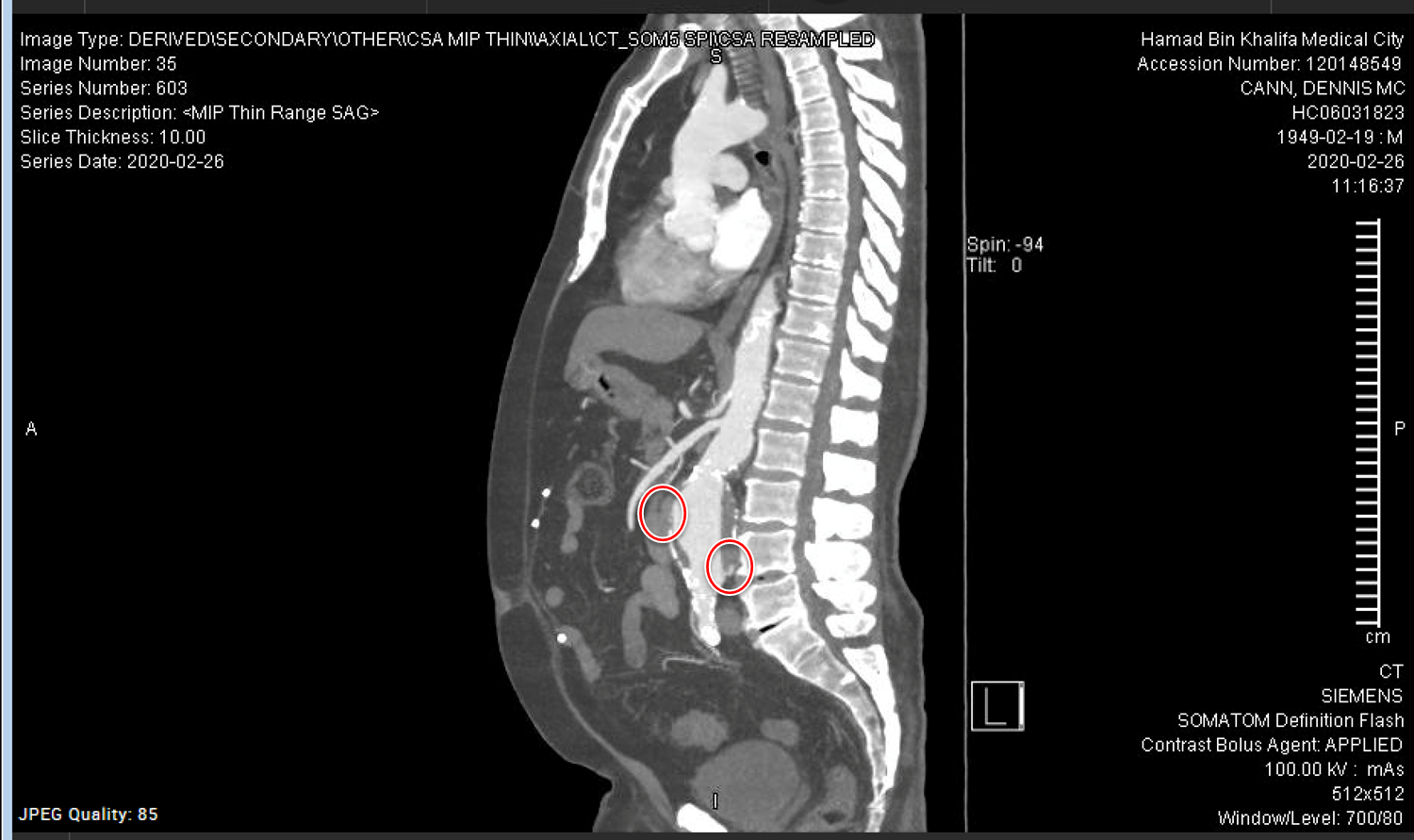 |

**FIGURE S2** Distribution of WSS parameters of TAWSS, OSI, ECAP, and RRT in contour plots for **all** ruptured fusiform high ILT aneurysm cases (patients 13, 14, 15, 18, 19, 20, and 21). The red circles indicate the clinician-defined rupture site in the CT data and the contour plot area corresponding to the same location.
